# Polyphenolic extract of InsP 5-ptase expressing tomato plants reduce the proliferation of MCF-7 breast cancer cells

**DOI:** 10.1371/journal.pone.0175778

**Published:** 2017-04-27

**Authors:** Mohammad Alimohammadi, Mohamed Hassen Lahiani, Diamond McGehee, Mariya Khodakovskaya

**Affiliations:** 1 Department of Biology, University of Arkansas at Little Rock, Little Rock, Arkansas, United States of America; 2 Institute of Biology and Soil Sciences, Far-Eastern Branch of Russian Academy of Sciences, Vladivostok, Russia; University of Alabama at Birmingham, UNITED STATES

## Abstract

In recent years, by extensive achievements in understanding the mechanisms and the pathways affected by cancer, the focus of cancer research is shifting from developing new chemotherapy methods to using natural compounds with therapeutic properties to reduce the adverse effects of synthetic drugs on human health. We used fruit extracts from previously generated human type I InsP 5-ptase gene expressing transgenic tomato plants for assessment of the anti-cancer activity of established genetically modified tomato lines. Cellular assays (MTT, Fluorescent microscopy, Flow Cytometry analysis) were used to confirm that InsP 5-ptase fruit extract was more effective for reducing the proliferation of breast cancer cells compared to wild-type tomato fruit extract. Metabolome analysis of InsP 5-ptase expressing tomato fruits performed by LC-MS identified tomato metabolites that may play a key role in the increased anti-cancer activity observed for the transgenic fruits. Total transcriptome analysis of cancer cells (MCF-7 line) exposed to an extract of transgenic fruits revealed a number of differently regulated genes in the cells treated with transgenic extract compared to untreated cells or cells treated with wild-type tomato extract. Together, this data demonstrate the potential role of the plant derived metabolites in suppressing cell viability of cancer cells and further prove the potential application of plant genetic engineering in the cancer research and drug discovery.

## Introduction

Cancer is one of the leading causes of death in humans. Scientific advances in recent years and the use of chemoprevention therapy has led to a significant reduction in death rates for different types of cancers [1–3]. Recently, natural compounds with cancer preventive properties have been more widely used in cancer therapy [4]. Natural compounds with antioxidant activity can be categorized into three major groups: compounds that can directly inhibit cell proliferation, compounds that affect tissues outside the cancer cells, and immune-stimulating compounds [5]. Epidemiological studies have shown a positive correlation between the long-term consumption of fruits and vegetables containing naturally occurring antioxidants with a reduced risk of several types of cancer [6–10]. One of such naturally occurring antioxidants are polyphenols that can be found in various amounts in many types of fruits and vegetables [11–13]. They can be classified into two main groups according to their chemical structure: flavonoids and non-flavonoid compounds. These compounds are particularly valuable because of their high antioxidant activity [14–16]. Several clinical studies indicate that dietary intake of flavonoids and some other phenolic compounds such as caffeic acid and chlorogenic acid can significantly reduce the risk of multiple types of cancer including breast, lung, prostate, and pancreatic cancers [17–20]. Studies have also shown that the use of dietary phenolic compounds can have better preventive and therapeutic results compared to the common synthetic drugs used for cancer treatment since these natural compounds demonstrate less toxicity compared to synthetic chemo-preventive medicines [21].

Dietary flavonoids and other important phenylpropanoids naturally exist in plants. A good example of the commonly used crop plants with a high content of phenolic compounds is tomato (*Solanum lycopersicum*) [22]. Consumption of tomato has preventive and therapeutic effects on several types of diseases, including cancer [23]. The observed anti-cancer effects of tomato are mainly related to the properties of phenolic compounds that allow them to bind to or interact with a wide range of molecules, affect cell signaling processes, or even serve as a signaling molecule [24–27].

**Fig 1:**
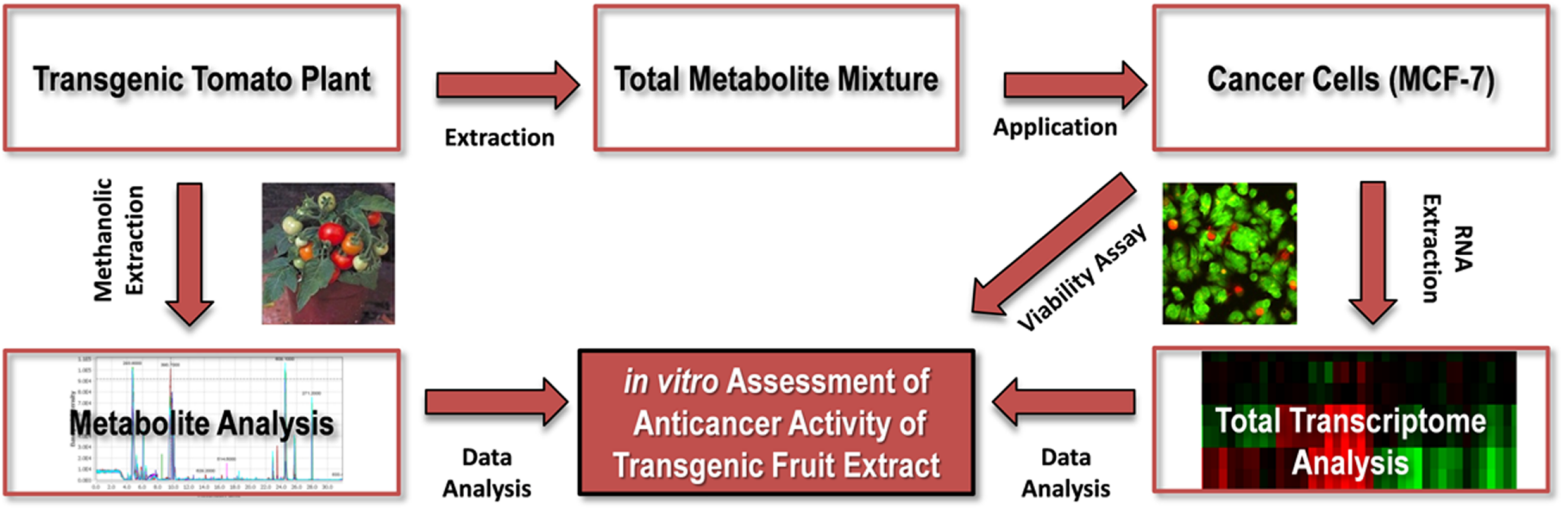
Overview of applied research strategy. Total metabolites of transgenic tomato fruits were analyzed for their potential anti-cancer properties by application of the total metabolite mixture to MCF-7 cancer cells followed by cell viability assays and transcriptome analysis of the cancer cells. The total tomato wild-type and transgenic fruit extracts were scanned for the potential metabolite with antioxidant and anti-cancer properties by using LC-MS.

Several attempts have been made to improve the level of health promoting compounds in tomato through conventional breeding techniques as well as genetic engineering tools [28,29]. We recently generated transgenic tomato lines with increased biosynthesis of antioxidants such as lycopene, vitamin C and several flavonoids [30]. Particularly, the transgenic lines were generated by overexpression of InsP 5-ptase gene which affects the phosphoinositol stress signaling pathway through changes in the metabolism of InsP_3_, the key metabolite of the phosphoinositol pathway [31]. We also reported that the increase in metabolism of InsP_3_ in transgenic plants positively affects the biosynthesis of several flavonoids, such as chlorogenic acid and rutin, by changing the expression level of the main components of the light-signaling pathway that is linked to secondary metabolism in plants [32]. The observed increase in biosynthesis of phenolics and other secondary metabolites with antioxidant properties in InsP 5-ptase overexpressing transgenic plants suggest an increase in health beneficial properties of these transgenic tomato plants. Despite the obvious potential of the genetically enhanced crop plants with enhanced nutraceutical value, general concerns regarding consumption of food products containing genetically modified (GM) ingredients significantly limits the use of GM crops in medicine. In such circumstances, the extraction of desirable pharmaceuticals from GM crops can serve as an alternative approach to the direct consumption of GM crops [33–35]. These compounds can then be purified and used in medicine as drugs or supplements. Here, we tested the anti-cancer activity of the total metabolite extract containing flavonoids and other phenolic compounds from InsP 5-ptase expressing tomato fruits *in vitro*. Anti-proliferative effects of extracts obtained from transgenic fruits on breast cancer cell line (MCF-7) were documented by a number of standard assays including cell viability assay, cell morphological analysis, and flow cytometry. Total transcriptome analysis of cancer cells treated with a mix of metabolites extracted from InsP 5-ptase fruits suggested possible pathways involved in anti-cancer effects of applied extracts. To identify metabolites that may play a role in the anti-proliferative activity of InsP 5-ptase fruit extracts, we analyzed and compared extracts from wild-type tomato fruits (control) and extracts from transgenic fruits using LC-MS as a powerful and modern metabolomics tool. LC-MS data confirmed the up-regulation of a number of phenolic compounds with strong anti-proliferative potential in InsP 5-ptase fruit extracts. The design of our study is shown in Fig 1.

## Materials and methods

### Plant growth conditions

InsP 5-ptase tomato lines were generated and described in details in our previous publication (30). Tomato (cv. Micro-Tom) seeds of control lines (wild-type, empty vector control, and transgenic lines (lines 6 and 7) were germinated in pots containing a combination of 75% Sun Gro Redi-earth ‘Plug and Seedling’ Mix (Sun Gro Horticulture, Bellevue, WA) and 25% sand. The seeds were germinated in a growth chamber under high-light conditions (800 μmol m^-2^ s^-1^) with intervals of 16 h light (25°C) and 8 h dark (22°C). The red fruits were collected between 6 to 8 weeks of growth under controlled environment and exposure to high-light. The red tomato fruits were immediately frozen in liquid nitrogen after harvest and stored at -80°C or immediately used in the experiment. For phenolic extraction, fruit samples were immediately lyophilized and stored in the dark environment at room temperature before being used in the experiment.

### Total phenolic compounds extraction and quantification

Total phenolics were extracted based on the method described by Ainsworth and Gillespie (2007) [36]. The colorimetric assay works based on the transfer of electrons in alkaline medium from phenolic compounds to phosphomolybdic/ phosphotungstic acid complexes. A three step sequential aqueous/methanol extractions method was used to extract Polyphenols, hydroxycinnamates, flavonoids, and their glycosides from lyophilized red tomato fruits. One milliliter of the methanol/water (2:1) solution was mixed with 100 mg of each fruit sample after which the samples were vortexed for 30 minutes. Next, the sample extracts were centrifuged at 10, 000 g for 5 minutes at 4°C and the supernatant was collected. One milliliter of fresh extraction solution was added to the pellet and the above extraction process was repeated twice and at the end of each extraction, the supernatant was collected. It is important to protect the samples from light throughout the extraction process. Folin-Ciocalteau micro method as described by Slinkard et al. (1977) was used to measure the concentration of the phenolic compounds in the extraction solution [37]. Twenty microliters of the tomato extract were diluted in 1.58 ml of H_2_O and 100 μl of the Folin-Ciocalteau reagent was added to the solution. The mixture was vortexed for 5 minutes after which 300 microliters of sodium carbonate solution (250 μg/ml) were added to the reaction, mixed and incubated for 30 minutes at 4°C. Following the incubation period, absorbance was read at 765 nm against the blank. A galic acid calibration curve (0–1 g/L) was used as standard and the flavonoid concentrations were expressed as galic acid equivalents.

### Fruit tissue preparation for LC-MS analysis

Whole, intact tomatoes were pulverized using a mortar and pestle with liquid nitrogen to keep tissue frozen. Approximately 20 mg of frozen tissue powder was then extracted using 80% methanol by using a bead beater for each of the three technical replicates for each line. Samples were then vortexed, sonicated, and centrifuged. The supernatant was filtered through 13 mm syringe filters into microcentrifuge tubes and dried overnight in a 45°C vacufuge. Samples were then stored at -80°C for future use. Extraction solution (80% methanol) was used to reconstitute samples to equal volumes. Samples were vortexed and sonicated to ensure all residues had dissolved before they were centrifuged. The supernatant was transferred to a labeled autosampler vial and analyzed immediately.

### Chromatography and mass spectrometry

Samples were analyzed using a Grace C-18 (Grace Davison Discovery Sciences, USA) reverse phase column on an Agilent 1100 series LCMS (Agilent Technologies, Waldbronn, Germany) equipped with a G1379A degasser, G1312A binary pump, G1329A autosampler, G1316A column oven, G13158B diode array detector, and G2445C MS. The aqueous phase was acidified HPLC-grade water (0.05% formic acid), and the organic phase was HPLC-grade methanol. Ten microliter injections were pumped at 0.6 mL/min with the following elution gradient: 0–2 min, 5% B; 2–22 min, 75% B; 22–27 min, 75% B; 27–28 min, 5% B; 28–32 min. A five-minute wash was included after each sample run. Negative-mode electrospray ionization was used to detect the metabolites by utilizing a trap mass spectrometer scanning of 100–1500 m/z. The target was set at 10,000 and maximum accumulation time at 100.00 ms with two averages. *Chemstation* software (http://www.agilent.com/en-us/products/software-informatics/massspec-workstations/lc-ms-chemstation-software), provided with the Agilent machine used to analyze samples and collect data was used to convert files to netCDF format. Further conversion to mzXML format was completed with msConvert (http://proteowizard.sourceforge.net/tools.shtml) [38]. Files were then loaded into MZmine software (http://mzmine.github.io/) and processed [39]. The Kyoto Encyclopedia of Genes and Genomes (KEGG) database, available at http://www.genome.jp/kegg/tool/map_pathway1.html, was used for tentative online compound identification and was completed through MZmine using the gap-filled peak list [40]. Further statistical analysis was carried out by uploading the identified peak list to Metaboanalyst (http://www.metaboanalyst.ca/) for analysis and by comparison to publications [41–43]. More information regarding data structure can be found in S4 Fig.

### Cell culture conditions

MCF-7 breast cancer cells (ATCC) were seeded in T-75 culture flasks (Thermo Scientific) and maintained in Dulbecco’s modified Eagle‘s medium (DMEM) media, supplemented with 10% fetal bovine serum (FBS), 100 U/ml penicillin and 100 U/ml streptomycin. The culture plates were maintained at 37°C with 5% carbon dioxide. The medium was changed every two days, and the cells were passaged at 80% confluency before the experiment.

### MTT assay

Cells were divided into six groups: blank group (no cells), control group (no treatment) and four experimental groups (WT, EV, L6 and L7 lines extract treatments). Cells were seeded in 96-well plates 24 hours prior the experiment at the density of 10^4^ cells/well. The next day, the medium was changed and metabolite extract (34 μg/μl was supplemented to the fresh medium. The cells were incubated 24 hours with the medium containing the metabolite extract. After the incubation period, 3-(4,5-dim ethylthiazol-2-yl)-2,5-diphenyl-2H-tetrazolium bromide (MTT) was then added to each well at the concentration of 5 mg/ml. The cells were incubated for 4 hours, after which the supernatant was replaced with 200 μL of dimethyl sulfoxide (DMSO). The absorption was measured at 570 nm using a micro-plate reader. The results were presented as OD 570–620 using the following formula: MTT OD 570–620 = (Mean A 570–560)—(Mean A of Blank) / (Mean A Negative Control)—(Mean A of Blank). The results are based on two independent experiment with each experiment consisting of 3 technical replicates.

### Double immuno-staining and microscopy

MCF-7 were seeded into each well of Lab-Tek 2 chamber slide (Thermo Scientific Nunc. NY) and incubated for 4 hours at 37°C (5x10^5^ cells in each chamber) in a humidified, 5% carbon dioxide atmosphere to attach. Cells were divided into five groups: control group (no treatment) and four experimental groups (WT, EV, L6 and L7 lines extract treatments). Total metabolite extract (34 μg/μl) was then added to fresh DMEM medium and applied to the wells and incubated for 24 hours. After incubation, cells were washed once with phosphate buffer saline (PBS) and stained with 1% Acridine orange/Ethidium bromide solution in PBS for 1 minute. Chambers were then washed two times with PBS after which slides were detached from the chamber and air dried. Images were then taken by fluorescence microscopy. A Nikon Eclipse 90i microscope equipped with a 12V-100W halogen lamp, external transformer, fly-eye lens built-in and NCB11, ND8, ND32 filters, was used to visualize the stained samples. The following Nikon filters were used: barrier filter BP365, reflector filter FT 395 and exciter filter LP395. Live/dead cells were counted using Image J software. Live cells fluoresce green (FITC/green) and dead cells fluoresce red/orange (Texas Red/red).

### Flow cytometry analysis

Cells of MCF-7 breast cancer cell line were seeded in 6-well plates at a density of 5 x10^5^ cells per well and incubated for 24 hours at 37°C in an incubator with 5% carbon dioxide to attach. After the initial seeding, the cells were incubated with fresh medium containing 34 micrograms per microliters of extract and were incubated for 24 hours at 37°C. Next day, cells were trypsinized and collected in 2 ml tubes. Samples were centrifuged for 15 minutes at 1,300 rpm at 37°C. The supernatant was discarded and the cells were resuspended by addition of cold PBS. Samples were briefly vortexed, transferred to Flow Cytometry tubes, and then placed on ice. Components of YO-PRO kit (Life Technologies) were used for labeling the samples. One sample was kept as a control (without label). One microliter of the YO-PRO -1 stock solution (Component A) was added directly to the mixture in each tube followed by one microliter of the propidium iodide (PI) stock solution (Component B). Labeled tubes were incubated on ice for 30 minutes. Cells were analyzed by flow cytometry using BD LSRFortessa Cell Analyzer (BD biosciences, CA) (to detect green (YO-PRO-1) and red (propidium iodide) signals.

### Gene expression analysis using microarray (Affymetrix platform)

Breast cancer cell line MCF-7 cells were seeded in 6 well plates at a concentration of 10^6^ cells/well, 24 hours prior to the experiment. Afterwards, cells were washed two times with warm media and were incubated for 24 hours with fresh medium containing tomato extracts from different plant lines (WT, EV, L6, L7) at a concentration of 34 μg/μl. RNeasy Mini Kit (Qiagen Sciences, Maryland, USA) was used to isolate RNA samples with modification of standard Qiagen protocol. Cells were disrupted briefly by using Trizol reagent (Ambion, Grand Island, NY), and total RNA was extracted by using chloroform extraction method. After the RNA purification, on-column DNA digestion using the RNase-free DNase Kit (Qiagen Inc. Valencia, CA) was used to remove the residual DNA. The purity of RNA samples was confirmed by electrophoresis and the concentration was quantified by using Nanodrop spectrophotometer (Thermo Scientific. Wilmington, DE). *Affymetrix Human Genome Arrays* were used as the microarray platform. Biotinylated cRNA targets were synthesized by Affymetrix IVT Express target labeling assay as specified in the Affymetrix GeneChip Expression Analysis Technical Manual. Hybridization reactions to the Affymetrix Human GeneChips were carried out by Expression Analysis, Inc. (Durham, NC).

### Statistical analysis

The cell viability data were analyzed by Tukey’s test and expressed as mean ±S.D. by which the significant differences (*P* value < 0.05) between groups were determined. To analyze the microarray raw data, column-wise normalization using a reference sample (control)was applied. The resulting data was then visualized using Multi Experiment viewer (Mev). The data was further analyzed by ANOVA and Tukey test with a p-value of 0.001 while assuming variance between variables are equal. The genes with significantly different expression were clustered by hierarchical clustering. In addition, all the known and unknown genes with changes in expression were functionally analyzed using the Database for Annotation, Visualization and Integrated Discovery (DAVID) v6.7 and PANTHER for gene classification. Microarray data were deposited in GEO database (GEO number: GSE94548).

## Results

### Effect of InsP 5-ptase overexpression in tomatoes on total phenolic content in transgenic fruits

**Fig 2:**
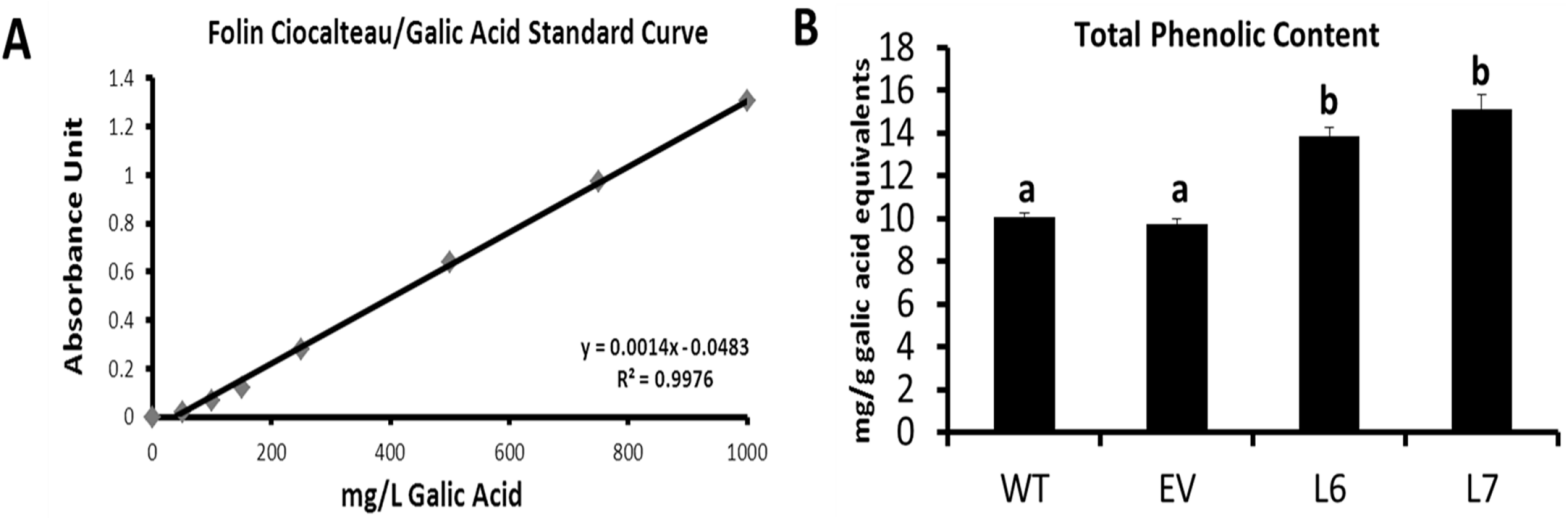
Total phenolic content in mature (red) InsP 5-ptase expressing and wild-type tomato fruits. (A) Standard curve using galic acid (0–1000 mg) as a standard reagent. (B) Total phenolic content in the mature tomato fruits of InsP 5-ptase transgenic lines (L6 and L7) and control lines (WT and EV). The total phenolic content is expressed as mg/g galic acid equivalent. The different letters (a,b) means statistically different groups (p<0.01) using Tukey Post-ANOVA test. Error bars represent standard error value.

InsP 5-ptase overexpressing (two independent transgenic lines) and control (two control lines) tomato fruits were tested for their total content of metabolites with phenolic nature. Results of the experiment demonstrated that the fruits of transgenic tomato lines contain more phenolic compounds (37% for L6, 50% for L7) compared to control lines (Fig 2). These results confirm that InsP 5-ptase expressing tomato fruits (L6, L7) accumulate higher levels of phenolic compounds compared to the control fruits (WT, EV).

### Effect of InsP 5-ptase overexpression on total metabolome of transgenic tomato fruits

**Fig 3:**
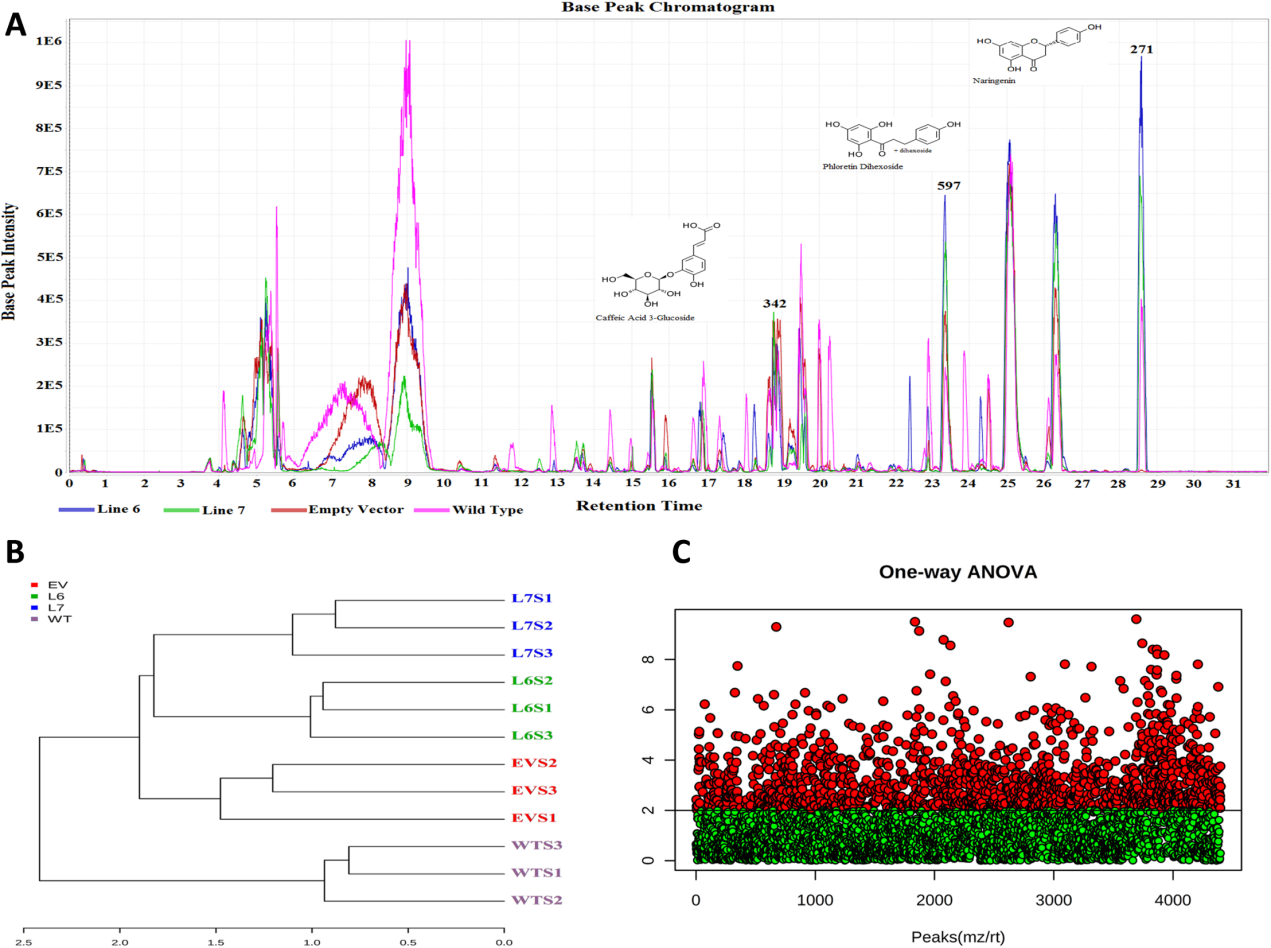
Analysis of metabolome of wild-type (WT), empty vector control (EV) and InsP 5-ptase expressing tomato fruits (L6, L7) using LC-MS. (A) Chromatogram built by MZmine. The chromatogram includes one fruit sample from each line analyzed: Line 6 (blue), Line 7 (green), Empty Vector (red), and Wild-Type (pink). Multiple compounds have been highlighted. Some minor retention shifts were corrected by the program. (B) Dendrogram created by Metaboanalyst from the list of cursorily identified peaks, showing the clustering of samples. (C) Metaboanalyst completed an ANOVA analysis on all samples with a p value < 0.01. Green dots are a representation of metabolites that were not significantly different. Red dots are a representation of metabolites that were significantly different between at least two samples.

Initially, LC-MS analysis of the mature control and transgenic tomato fruits resulted in the identification of over 7000 metabolites (Fig 3). The chromatogram is shown as Fig 3A was built by the MZmine software and gives a cursory visual interpretation of initial data from the LC-MS.

**Fig 4:**
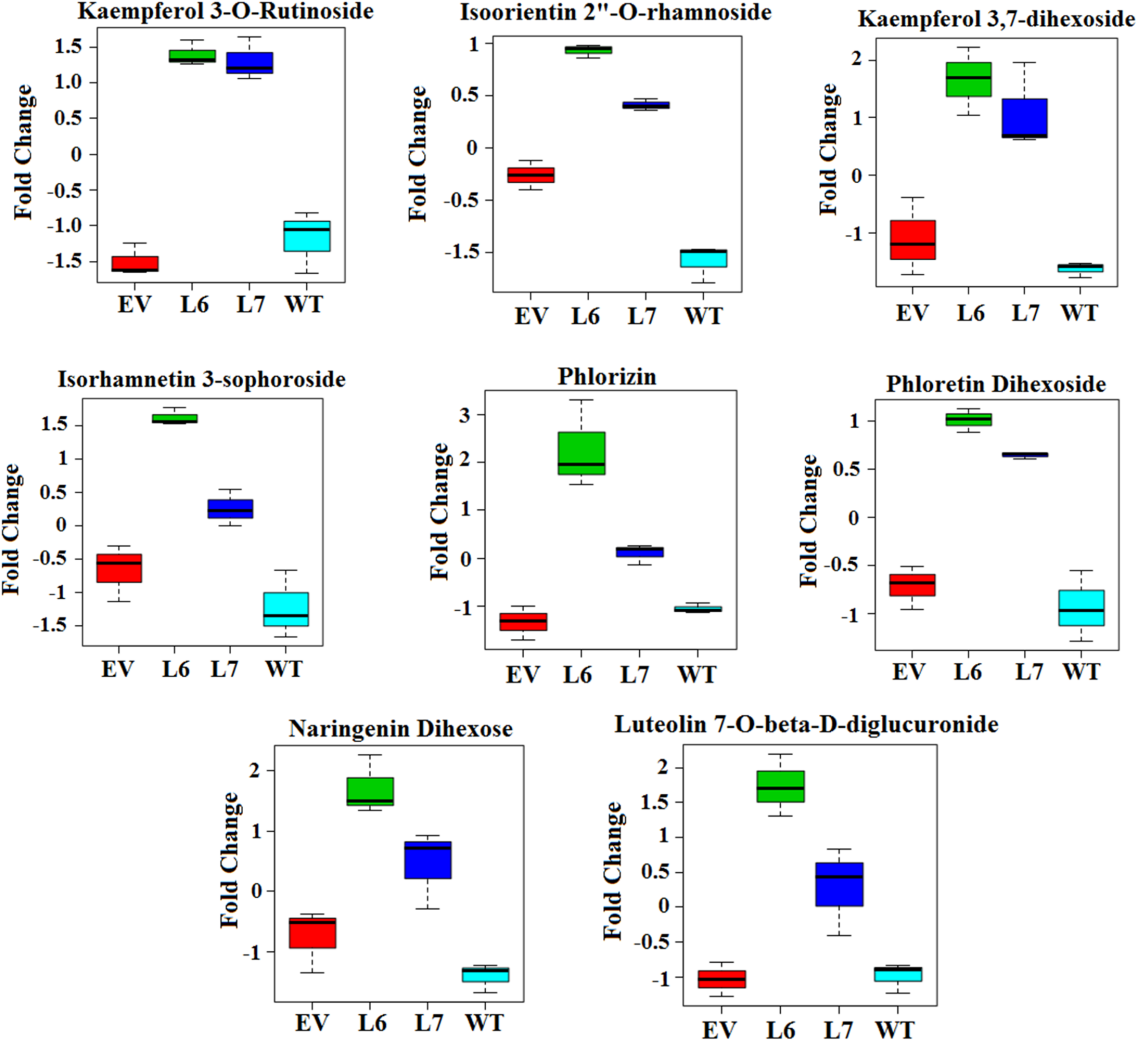
Relative differences in concentrations between control (WT, EV) and transgenic tomato samples (L6, L7) for eight identified flavonoid metabolites. The relative increase of concentration was based on average intensity fold change between samples. Separate images were created with Metaboanalyst using data generated by LC-MS.

One sample from each line was chosen for simplicity of chromatogram presentation. Results showed that there is a good alignment of peaks and that the peaks observed are well defined. As the next step, MZmine was used to create a list of peaks that were suitable for comparison to the KEGG database for the initial identification of compounds. From the initial list of over 7000 possible metabolites, 4047 (55%) were cursorily identified using the KEGG database through MZmine. Identification of metabolites is an ongoing challenge due to the lack of well-developed databases, especially for plants [44]. Additionally, comparison to databases is generally unreliable due to the poor reproducibility of retention time for LC systems between laboratories. However, several successful attempts to identify the tomato metabolome by LC-MS have been reported, leading to the development of other databases, like MotoDB (http://www.ab.wur.nl/moto/) and KNApSAcK (http://kanaya.naist.jp/knapsack_jsp/top.html) [45,46]. The dendrogram in Fig 3B demonstrated that Empty Vector and Wild-Type control groups were more related to each other than to transgenic lines, as expected. Additionally, each transgenic line (L6, L7) was more related to its counterpart than to the Wild-Type and Empty Vector control sample groups. These results indicated that extraction procedures were successful, LC-MS conditions were stable, and the integration of vector (EV) without foreign InsP 5-ptase gene into the tomato genome did not significantly change the metabolome. The one-way ANOVA in Fig 3C highlighted (in red) over 2000 metabolites (29%) between the lines that were significantly different at an alpha level of 0.05. Fisher’s test determined 117 (5.4%) metabolites were up-regulated in both transgenic lines, seen in S1 Table. Using online databases and publications, 35 (30%) of the 117 metabolites were identified in S2 Table. 11 (38%) of those 35 metabolites were found to have a typical chemical flavonoid structure [46–48]. The total 35 tentatively identified compounds can be seen in along with their respective pathways if such information was available. Other metabolite types included amino acids, carbohydrates, and nucleotides, all of which are extractable using a phenolic method [48]. Finding upregulated flavonoids is interesting because it has been reported that strong effects on cancer cell mortality are associated with the flavonoid nature of several tested metabolites [49–52]. Many of the detected metabolites, such as narigenin dihexose and isoorientin 2”-O-rhamnoside, are glycosides, the preferred storage form of flavonoids for plants [52]. Relative differences between control and transgenic samples for eight identified flavonoid metabolites can be seen in Fig 4. Here we present fold changes because the LC-MS machine used was not suited for explicit quantitative analysis. The level of naringenin dihexose, for example, was almost 2-fold higher in Line 6 and almost a full fold higher in Line 7 tomatoes compared to Empty Vector and Wild-Type control lines. Increased levels of flavonoids, such as naringenin dihexose, suggested that pathways associated with secondary metabolism and, therefore, production of secondary metabolites would be affected by the genetic alteration.

Over 20 affected pathways were identified via database comparison, some of which are shown in The metabolic pathways and the biosynthesis of secondary metabolites displayed the highest number of identified compounds with 220 and 124 respectively. Table showing affected pathways was created to highlight pathways in which identified compounds are involved and their relative regulation observed through LC-MS. Overall, we noticed that the most affected pathways in transgenic fruits are metabolic pathways and the pathways for biosynthesis of plant secondary metabolites, as expected. Observed massive up-regulation of flavonoid metabolites in transgenic InsP 5-ptase fruits may indicate the potential of InsP 5-ptase fruit extract as an inhibitor of the proliferation of cancer cells. To test our hypothesis, we took a classical *in vitro* approach and investigated viability and proliferation of breast cancer cells (MCF-7 line) after treatment with extracts from control and InsP 5-ptase fruits.

### Effect of total metabolite extract from InsP 5-ptase expressing transgenic tomato fruits on the viability of cancer cells

**Fig 5:**
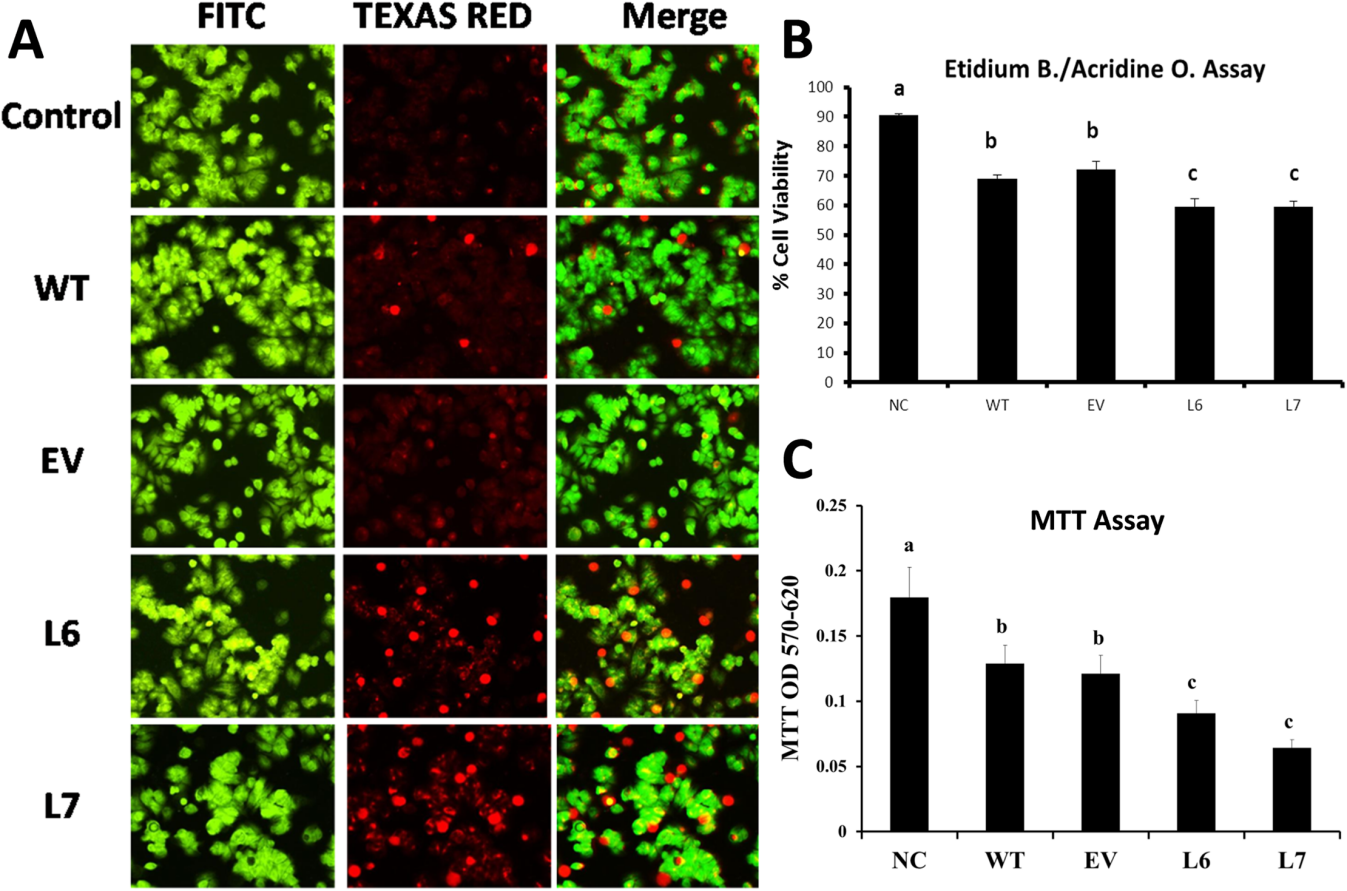
Assay of cell viability of MCF-7 breast cancer cells treated with tomato extracts originated from control (WT, EV) and transgenic fruits (L6, L7). (A) Fluorescent microscopy images demonstrating the Ethidium bromide/Acridine orange (EB-AO) staining assay on MCF-7 cancer cells. The cells were treated with total metabolite extract (1μg/ml) from transgenic (L6 and L7), and control (WT and EV) tomato fruits. The incubation time was 24 hrs at 37°C with 5% CO_2_. The green fluorescence demonstrates the nucleus of the viable cells and the red fluorescence represents the nucleus of the apoptotic cells. The image is representative of the two independent experiments. (B) Statistical analysis of the EB-AO staining assay demonstrating the viability of the MCF-7 cells after incubation with total metabolite extract of tomato fruits. (C) MTT cell viability assay performed on MCF-7 cells using the same conditions to confirm the results of (EB-AO) assay. The different letters (a,b,c) means statistically different groups (p<0.01) using Tukey Post-ANOVA test. Error bars represent standard error value.

Results of cell viability assay demonstrated that incubation with the phenolic extract obtained from fruits of control lines (WT, EV) reduced the cell viability of MCF-7 cancer cells to 70 percent of the level of control cells (Fig 5). Incubation of the cells with the 1 μg / ml of extract from InsP 5-ptase transgenic lines decreased the cell viability even more, to as low as 50 percent for line 6 (L6) and 30 percent of the level of control cells for line 7 (L7) (Fig 5C).

Acridine orange—Ethidium bromide double staining method was used to confirm the results of the MTT assay and to visualize the cell viability of MCF-7 cells in response to treatment with phenolic extract. MCF-7 cells were incubated with total phenolic extract of control lines (WT and EV) as well as transgenic lines (L6 and L7). After the incubation period, cells were stained using Acridine orange/Ethidium solution. Dead and live cells were differentiated by utilizing a fluorescent microscope imaging system. It was found that there were fewer living cells in the samples that were incubated with a phenolic extract obtained from transgenic lines (L6, L7) compared to control lines (WT, EV) (Fig 5A). The further statistical analysis confirmed that the number of live MCF-7 cells were decreased to 60 percent in response to incubation with phenolic compounds derived from transgenic lines in comparison with non-treated control cells. This is a ten percent decrease in cell viability compared to the incubation of the cells with the fruit extract of wild-type tomato plants (Fig 5B).

### Analysis of the effect of total fruit extracts of InsP 5-ptase expressing transgenic tomato fruits on cell proliferation of cancer cells

**Fig 6:**
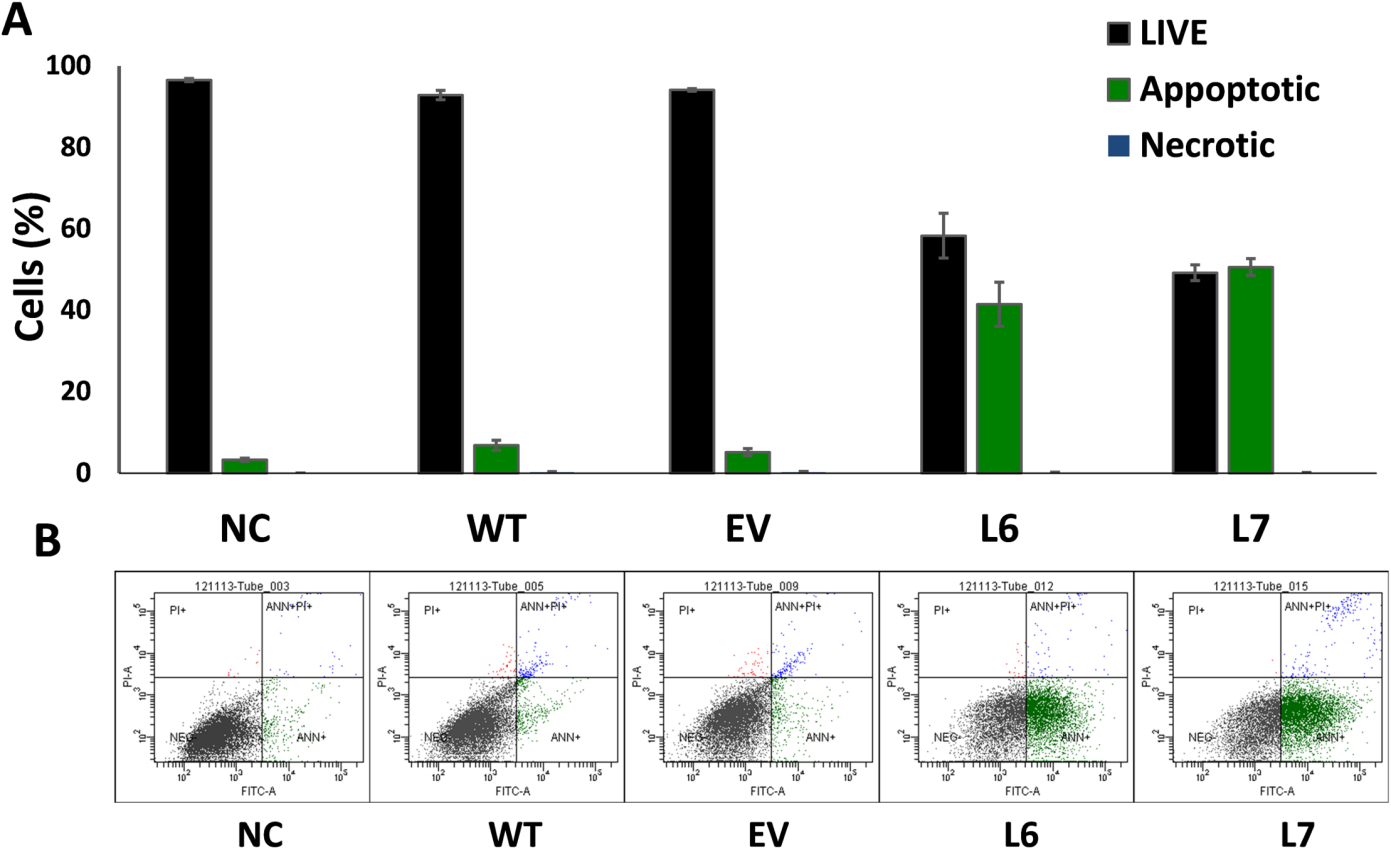
Data of flow cytometry analysis of MCF-7 breast cancer cells treated with tomato extracts originated from control (WT, EV) and transgenic fruits (L6, L7). (A) Flow Cytometry analysis on MCF-7 cancer cell line after treatment with total metabolite extract of transgenic and control fruits. The results are presented as a percentage of live, necrotic and apoptotic cells (100% total). The cells treated with 1μg/ml of the total metabolite extract of control (WT and EV) and transgenic (L6 and L7) tomato fruit are compared to the non-treated cells (NC). Three biological replicates were used for this experiment. (B) Flow Cytometry analysis graph for representatives of the non-treated MCF-7 cells and the cells treated with total metabolite extract of tomato fruits. The figures show live cells (bottom left quadrant), apoptotic cells (bottom right quadrant) and necrotic cells (top quadrants).

According to the results of our previous experiments, InsP 5-ptase transgenic tomato fruits accumulate up to 50 percent more phenolic compounds compared to the control fruits. This increase suggests higher antioxidant content and therefore, more antioxidant properties. The initial MTT assay experiment also shows that the incubation of the MCF-7 breast cancer cells with total metabolite extract of the transgenic fruits (L6, L7) reduces the cell viability in cancer cells compared to incubation with extract of the control lines (WT, EV) (S1 Fig). It is necessary to determine the nature and the mechanism of the cell death to gain a better understanding of the effect of the tomato extract on the cancer cells. For this reason, Flow Cytometry analysis was used to test the cell proliferation rate of the cancer cell lines (MCF-7) in response to incubation with total metabolite extract from fruits of both control and InsP 5-ptase expressing plants and to clarify the nature of cell death in these cell lines. The Flow Cytometry results obtained are in line with the data collected in previous experiments and confirm the role of the total metabolite extract in cell proliferation of breast cancer (MCF-7) cell lines (Fig 6). Flow Cytometry data demonstrate up to 50 percent decrease in cell viability of MCF-7 cells after incubation with total metabolite extract of transgenic tomato fruits (L6, L7) (Fig 6A).

### Microarray analysis of MCF-7 cells genes affected by the treatment of L6 and L7 tomato extract

In order to understand the mechanism by which transgenic tomato extract increase the proliferation of the cancer cells in transcriptional level, total transcriptome analysis was performed on MCF-7 breast cancer cells treated with transgenic tomato extract (L6 or L7) and breast cancer cells treated with extract of wild-type tomato (WT) or non-treated cancer cells (negative control).

**Fig 7:**
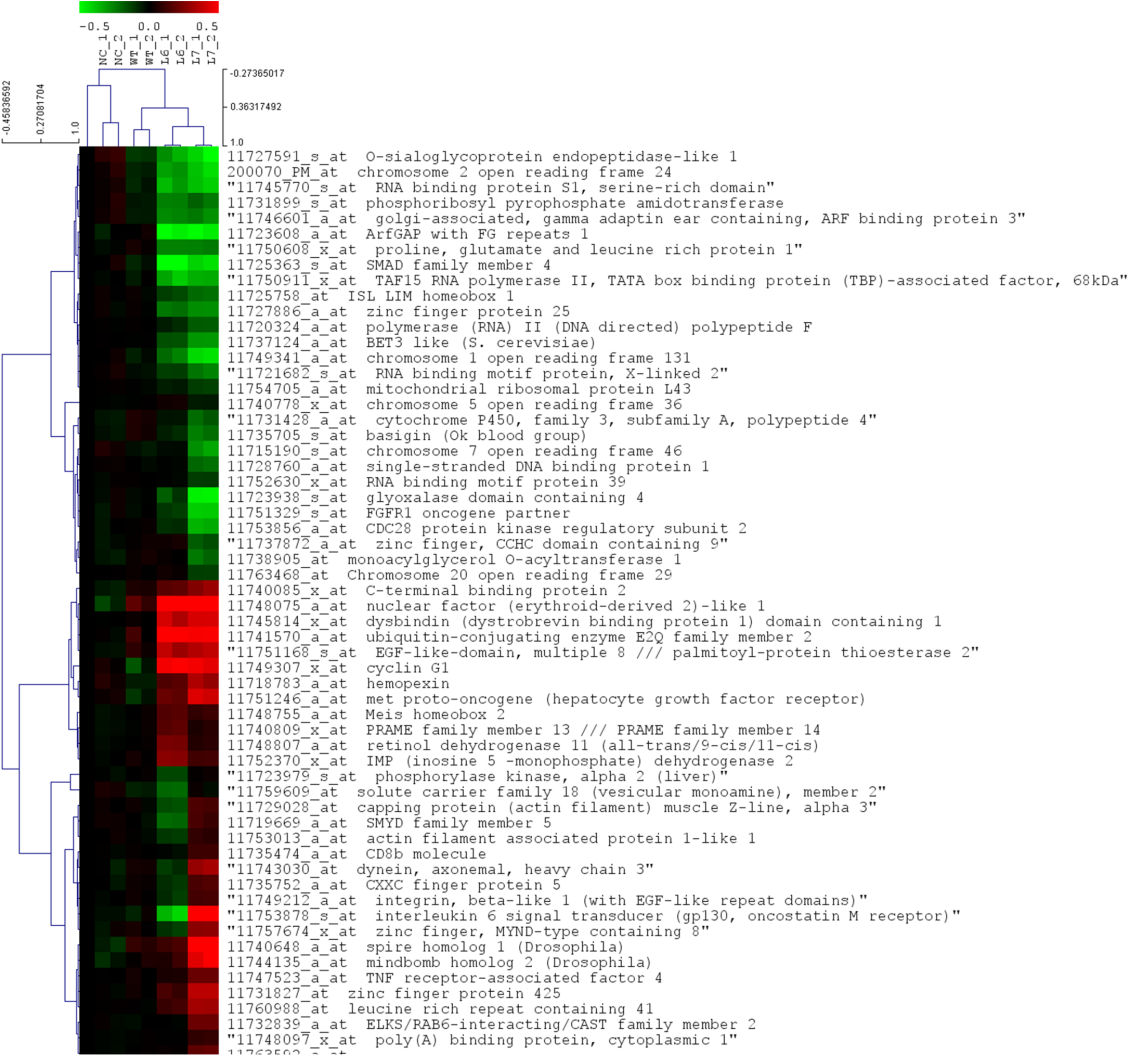
Microarray heat-map analysis of MCF-7 breast cancer cells treated with tomato fruit extracts originated from control and transgenic tomato lines. The analysis shows the genes that demonstrate changes in expression after treatment with total metabolite extract from transgenic lines (L6 and L7) and control line (WT) compared to non-treated cells (NC). The MCF-7 cells were incubated with the tomato fruits extracts (1μg/ml) for 24 hrs, after which the cells were harvested and the total RNA was extracted. The genes with significant change in expression were selected after statistical analysis using ANOVA statistical test using a *p*-value <0.001 and Tukey post-hoc test. Only the genes with identified functions are displayed. The range of fold change of identified genes is from -0.5 to 0.5.

The microarray analysis has shown that several genes were differently expressed in the cells treated with L6 or L7 tomato extracts compared to cells treated with wild-type tomato extract (Fig 7). ANOVA analysis and post-hoc analysis showed that 58 genes were differently expressed in the cells treated with L6 or L7 tomato extract compared to cells treated with an extract of wild-type tomato. A total of 28 genes were down-regulated in both L6 and L7 treated cancer cells, while 19 genes were up-regulated in both treatments. On the other hand, we found that 11 genes were down regulated in L6 treated cancer cells and up-regulated in L7 treated cancer cells. The gene ontology classification using DAVID software has shown that most of the differently expressed genes had trivial functions in cell transcription machinery. Examples of these functions included RNA splicing, mRNA processing, mRNA splicing, RNA recognition motif and nucleotide binding. Genes that contribute to the positive regulation of transcription from RNA polymerase II promoter including SMAD family member 4 and ISL LIM homeobox 1 were both down regulated in cancer cells treated with L6 or L7 tomato extract. On the other hand, genes responsible for the negative regulation of transcription including Meis homeobox 2 and C-terminal binding protein 2 were both up-regulated in L6 and L7 tomato extract treated cancer cells. Furthermore, genes responsible for RNA splicing and mRNA processing including RNA binding protein S1, serine-rich domain, polymerase (RNA) II (DNA directed) polypeptide F and RNA binding motif protein 39 were all down regulated in cancer cells treated with transgenic tomato extracts.

The cluster analysis, which was performed to group genes with a similar expression pattern, showed that genes from treated cancer cells (WT, L6, and L7) are clustered together. However, genes from cancer cells treated with extracts of L6 and L7 lines had a different sub-cluster than genes from cancer cells treated with wild-type tomato. All known up- and down-regulated genes were functionally analyzed using the PANTHER software for gene classification. Results revealed that the major biological processes affected during L6 or L7 treatments to cancer cells were involved in metabolic processes (29.9%) (S3 Fig). 90.9% of those genes had intracellular functions. The genes that contributed to the alteration of this processes were narrowed to a total of 8 genes responsible for transcription from RNA polymerase II promoter (S3 Table).

Quantitative real-time PCR analysis (qRT-PCR) was used to confirm the microarray results. The qRT-PCR was carried for the genes cyclin G1 and EGF-Like domain as representative of the genes with changes in expression level as indicated by microarray data in transgenic lines (L6, L7). The qRT-PCR results confirmed the up-regulation of both genes as shown by the microarray results (S2 Fig).

## Discussion

Tomato fruits are valuable source of multiple phenolic compounds that are known as antioxidant agents. The phenolic compounds such as caffeic acid, quercetin, naringenin, rutin and kaempferol have been extensively studied due to their various health benefits [30,53–55]. It is found that phenolic compounds can affect various steps of cancer development such as proliferation, inflammation, and invasion processes [54]. Flavonoids are a major group of phenolic compounds of tomato [56]. Multiple attempts have been made to enhance the accumulation of flavonoids in tomato to maximize the antioxidants properties of the tomato fruit [57].

Six years ago we generated transgenic tomato plants with a modified phosphoinositol pathway by overexpressing human type I InsP 5-ptase gene in tomato (30). InsP 5-ptase overexpression increased the metabolism of primary metabolites of the phosphoinositol pathway [32]. The transgenic lines were found to have a higher lycopene and phenylpropanoids content compared to wild-type plants as a result of the interaction between the phosphoinositol pathway and light signaling pathway that directly affects biosynthesis of secondary metabolites in plants [30,32]. The increase in the content of multiple phenolic compounds with antioxidant and anti-cancer properties suggested an increase in health promoting properties of the InsP 5-ptase transgenic tomatoes. In order to determine the effect of changes in metabolite content of transgenic tomato plants on cancer cells, multiple *in vitro* assays were performed after the application of extracts from wild-type and transgenic tomato fruits. The incubation of breast cancer cells (MCF-7) with total metabolite extract of InsP 5-ptase plants demonstrated the reduction in cell viability and proliferation in cancer cells compared to non-treated cells and cells incubated with wild-type tomato extract.

Changes in cell viability in cancer cells can be associated with various factors that control different pathways in cells. The Flow Cytometry analysis of MCF-7 cells incubated with InsP 5-ptase tomato extracts revealed that the induction of apoptotic pathways might play a significant role in the reduction of cell viability in the treated cancer cells. We suggest that the compounds with antioxidant properties in tomato extract can be involved in observed cell viability reduction of MCF-7 cells.

To identify the metabolites with the potential role in observed cell viability reduction in cancer cells from a wide range of metabolites of the tomato fruits, total metabolomics analysis was performed by using LC-MS (S4 Fig). Metabolomic analysis of the wild-type and transgenic tomato fruits using LC-MS revealed an increase in the concentration of several phenolic compounds with antioxidant properties in InsP 5-ptase transgenic tomato fruits. A number of these compounds were found to have therapeutic application and anti-cancer properties. One of such compounds that we found to be accumulated in higher concentrations in transgenic fruits is naringenin. The accumulation of naringenin is increased in InsP 5-ptase transgenic tomato fruits compared to the fruits of wild-type tomato plants. Naringenin is found to induce cytotoxicity in MCF-7 cell lines [58]. It is also found to reduce cell growth in human breast cancer cells in a dose-dependent manner by inducing apoptosis [59]. Another important naturally occurring flavonoid that is increased in our transgenic tomato lines is kaempferol. Kaempferol treatment is known to have a significant effect in reduction of cell viability in the MCF-7 cells [60]. Studies have shown that kaempferol can inhibit the cell proliferation of breast cancer cells by activation of caspase cascade in a dose-dependent manner [61]. Phloretin is yet another natural phenolic compounds with anti-cancer properties that we found to have a higher concentration in transgenic InsP 5-ptase tomato fruits. Phloretin is found to be effective in multi-drug resistant breast cancer cell lines [62]. These cell lines express breast cancer resistance protein (BCRP) that confers resistance to anticancer agents. Flavonoids have similarities in structure with estrogen estradiol and other steroid hormones and therefore have the ability to bind to the estrogen receptor and mediate transcription of estrogen responsive genes [63]. Flavonoids with weak estrogenic activities such as naringenin, kaempferol and phloretin are found to induce cytotoxicity to the BCRP expressing cell lines [64].

The diverse nature of phenolic compounds in tomato metabolite extract and diverse mechanism of action of these compounds suggest the involvement of multiple pathways in the observed reduction of cell viability in MCF-7 cell lines. Our total transcriptome analysis revealed that total extract of transgenic tomato fruits could activate multiple biological processes that affect DNA and RNA metabolism, particularly, the transcription machinery and RNA polymerase II. The transcription machinery regulates a wide-range of activities in the cells including the induction of apoptotic pathway in cancer cells. Some flavonoids such as flavopiridol (synthetic flavonoid) are known cyclin-dependent kinase inhibitors and found to induce apoptosis in cancer cells by phosphorylation of RNA polymerase II related to cancer inducing genes [65,66]. In addition, other studies showed that dietary flavone affects the genes involved in cell cycle and apoptosis through modifications of mRNA level and by affecting the transcription machinery [67]. Data from our transcriptome analysis revealed the changes in expression of multiple genes involved RNA metabolism after exposure of the cancer cells with total tomato phenolic extract. These results indicate a possible correlation between the changes in the level of certain flavonoids in our tomato extract with the observed reduction in cell viability of MCF-7 breast cancer cells through regulation of RNA metabolism. The transcriptome analysis of our samples also revealed changes in the expression pattern of multiple homeobox genes such as ISL LIM Homeobox 1 and Meis Homeobox 2. Homeobox genes are a large and important family of regulators, and their expression is found to be deregulated during cancer [68–70]. In cancer studies, homeobox genes are considered cancer modulators because they can be associated in both oncogenesis and tumor suppression [71]. In other words, except a few cases, gain or loss of functions of homeobox genes is not the determinant factor for oncogenesis or tumor suppression. However, their deregulation can induce this process in cells. Our results demonstrated that the incubation of the cancer cells with total phenolic extracts of tomato, results in significant deregulation of multiple homebox genes. We suggest that changes in expression pattern of above mentioned homebox genes can potentially trigger and change the balance of gene expression in the cells in favor of cancer suppressing genes. Examples of involvement of flavonoids in the manipulation of gene expression through deregulation of homebox genes have been shown previously. As an example, curcumin, a phenolic compound found in many plant species is found to inhibit induction of NKX3.1 gene which is involved in initiation stage of the prostate cancer. NKX3.1 gene codes for a homeobox containing transcription factor that is crucial for the prostatic cancer development and curcumin is found to interrupt its function via antioxidant activity [72]. Due to the many activities of the homeobox families and their role in different stages of cell development, it is plausible that an increase in phenolic content in the cancer cells can induce changes in the regulation of homeobox genes involved in cancer suppression. Based on the generated and the previously published data, it appears that multiple mechanisms may be involved in the anti-cancer activity of metabolites with flavonoid structure derived from extracts of InsP 5-ptase expressing tomato fruits.

## Conclusion

Here, we showed that overexpression of the InsP 5-ptase in tomato fruits results in an increase the accumulation of the phenolic compounds in the fruits of the transgenic plants. We demonstrated that the extract of tomato fruits expressing InsP 5-ptase gene exhibited stronger anti-cancer activity *in-vitro* compared to fruit extract originated from wild-type. Our findings supported the significance of anti-cancer potential of transgenic InsP 5-ptase plants used as food source or source of natural medicinal compounds.

## Supporting information

S1 Fig MTT assay confirmation. The MCF-7 cells were incubated with different concentration (A: (0.1 μg/ml), B: (1 μg/ml), C: (2 μg/ml) and D: (3 μg/ml)) of the total metabolite extract of the control (WT, EV) and transgenic (L6, L7) tomato fruits. Samples were incubated for 2, 6, 18 and 26 hours of incubation after which were treated with MTT reagent. The absorbance was measured at 570 nm using a spectrophotometer. The cell viability was compared with non-treated cells. *, p<0.05 of L6 compared to control, ϯ, p<0.05 L7 compared to control.
(PDF)

S2 Fig qRT-PCR confirmation of microarray data. qRT-PCR was performed for cyclin G1 and EGF-LIKE domain as representative of the genes with changes in expression level as indicated by microarray data. The RNA samples were isolated and the cDNA was synthesized from the control cells and those treated with an extract of wild-type or transgenic tomato fruits (lines 6 and 7). The results of the qRT-PCR for both genes confirm the up-regulation of the genes with changes similar to the microarray results.
Methodological details: The transcript abundance was validated by using quantitative real-time PCR (qPCR) for two genes: Cyclin G1 and EGF-Like-Domain. The cDNA was synthesized by using SuperScript III First-Strand Synthesis System Kit (Invitrogen Inc.) and using Oligo(dT) primers. The synthesized cDNA was used as a template in the PCR reaction and gene-specific primers were used to amplify selected genes. The amplification of Cyclin G1 was carried by using a forward primer (5’- AGCTGAATGCCCTGTTGGAA -3’) and a reverse primer (5’- TGCAGTCATTCTGAGGCCAT -3’). The amplification of EGF-Like-Domain was carried by using a forward primer (5’- ATGAGACCGTCCTGGAGATGG -3’) and a reverse primer (5’- GCAGACCAGCCTCAGCAGAA -3’). The amplification of Actin was carried by using a forward primer (5’- TCGTCGCCCACATAGGAATC -3’) and a reverse primer (5’- TGCTCAGGGCTTCTTGTCCT -3’). The qRT-PCR analysis was carried using SYBR Green PCR master mix (Applied Biosystems, Inc.) using a C1000 Thermal Cycler equipped with a CFX96 Real-Time detection system (Bio-Rad, Hercules, CA, USA). The experiment was carried by using samples of three biological replicates and three technical replicates. Comparative count method was used to analyze and interpret the qPCR data. The extraction protocol was based on the method described by Ainsworth and Gillespie (2007) (1). The colorimetric assay works based on the transfer of electrons in alkaline medium from phenolic compounds to phosphomolybdic/ phosphotungstic acid complexes. This transfer is determined specifically at 765 nm by using spectrophotometry.
(PDF)

S3 Fig Pie chart of main biological processes affected in MCF-7 cancer cell line by treatment with extract from InsP 5-ptase tomato fruits. The major genes contributing to the highest impacted biological process was narrowed down using PANTHER classification software.
(PDF)

S4 Fig Overview of the metabolite analysis methodology. Raw data were converted to mzXML format by msConvert, were loaded into MZmine software for preprocessing. This software processes the raw data based on user-defined parameters specific to the machine used to collect data. A peak list for analysis was generated via mass detection, chromatogram building and deconvolution, normalization, alignment, and gap filling. Data were used to create the chromatogram and dendrogram seen in Fig 3A and 3B. Additionally, a list of over 7000 peaks that were suitable for comparison to the KEGG database was created for the initial identification of compounds. The KEGG database is one of the most comprehensive collections of metabolites to our knowledge. MZmine has an integrated search function that utilizes this database, which facilitates compound identification. Identification of metabolites is an ongoing challenge due to the lack of well-developed databases, especially for plants (45). Additionally, comparison to databases is generally unreliable due to the poor reproducibility of retention time for LC systems between laboratories. However, several successful attempts to identify the tomato metabolome by LC-MS have been reported, leading to the development of other databases, like MotoDB (http://www.ab.wur.nl/moto/) and KNApSAcK (http://kanaya.naist.jp/knapsack_jsp/top.html) (46,47). After initial compound identification, the generated peak list was uploaded to Metaboanalyst for statistical analysis. This software is hosted online and facilitates statistical analysis of data. It allows for normalization, sample group pooling, and data transformation andperforms a wide variety of statistical tests without requiring the user to learn any programming. Additionally, it allows for the development of visual interpretations of statistical analysis results, such as the one-way ANOVA in Fig 3C and the metabolite concentrations. The one-way ANOVA suggested over 2000 metabolites that were differently regulated between control and transgenic groups. Of these, Fisher's test identified 117 metabolites that were up-regulated in the transgenic lines. The up-regulated metabolites became the focus for a more in-depth, manual identification search using the KEGG database, KomicMarket database, KNApSAck database, and publications (47,53,54). This search yielded 35 metabolites that could be putatively identified and were up-regulated, 11 of which had a flavonoid structure. Finally, the KEGG database was reference for pathway analysis using the cursorily identified up-regulated compounds seen in S2 Table.
(PDF)

S1 Table List of the compounds identified by Metaboanalyst software and were found significantly up-regulated in transgenic fruits of lines 6 and 7. 35 compounds have been tentatively identified by comparison to either a database or publication, 11 of which are flavonoids. Where the table lists KEGG as a source, MZmine software was used to compare the peak list to the database for identification purposes before the peak list was uploaded to Metaboanalyst, which was used for statistical analysis after MZmine completed processing the raw data. Not all compounds are in the KEGG database, but KEGG IDs are listed for those that were found.
(PDF)

S2 Table Identified up-regulated compounds in transgenic tomato fruits. The list of KEGG IDs from S1 Table was entered into the KEGG Pathway search that resulted in the 35 tentatively identified compounds that were up-regulated in both transgenic lines (6, 7) and their associated pathways, if available. The most common pathways were metabolic and biosynthesis of secondary metabolites. The most common type of identified compound was in the flavonoid category.
(PDF)

S3 Table List of genes involved in transcription of RNA polymerase II promoter found by using PANTHER classification software analysis. The genes labeled in red are genes that were up-regulated in the cells after treatment with total metabolite extract of transgenic fruits (L6 and L7) and genes labeled green are the genes that were down-regulated after the same treatment.
(PDF)
