# Supplementary material for: Polyphenolic extract of InsP 5-ptase expressing tomato plants reduce the proliferation of MCF-7 breast cancer cells
Source: PLoS One. 2017 Apr 27;12(4):e0175778. doi: 10.1371/journal.pone.0175778 (PMC5407797; doi:10.1371/journal.pone.0175778)
Supplement: S1 Fig — The MCF-7 cells were incubated with different concentration (A: (0.1 μg/ml), B: (1 μg/ml), C: (2 μg/ml) and D: (3 μg/ml)) of the total metabolite extract of the control (WT, EV) and transgenic (L6, L7) tomato fruits. Samples were incubated for 2, 6, 18 and 26 hours of incubation after which were treated with MTT reagent. The absorbance was measured at 570 nm using a spectrophotometer. The cell viability was compared with non-treated cells. *, p<0.05 of L6 compared to control, ϯ, p<0.05 L7 compared to control. (PDF) [file pone.0175778.s001.pdf]

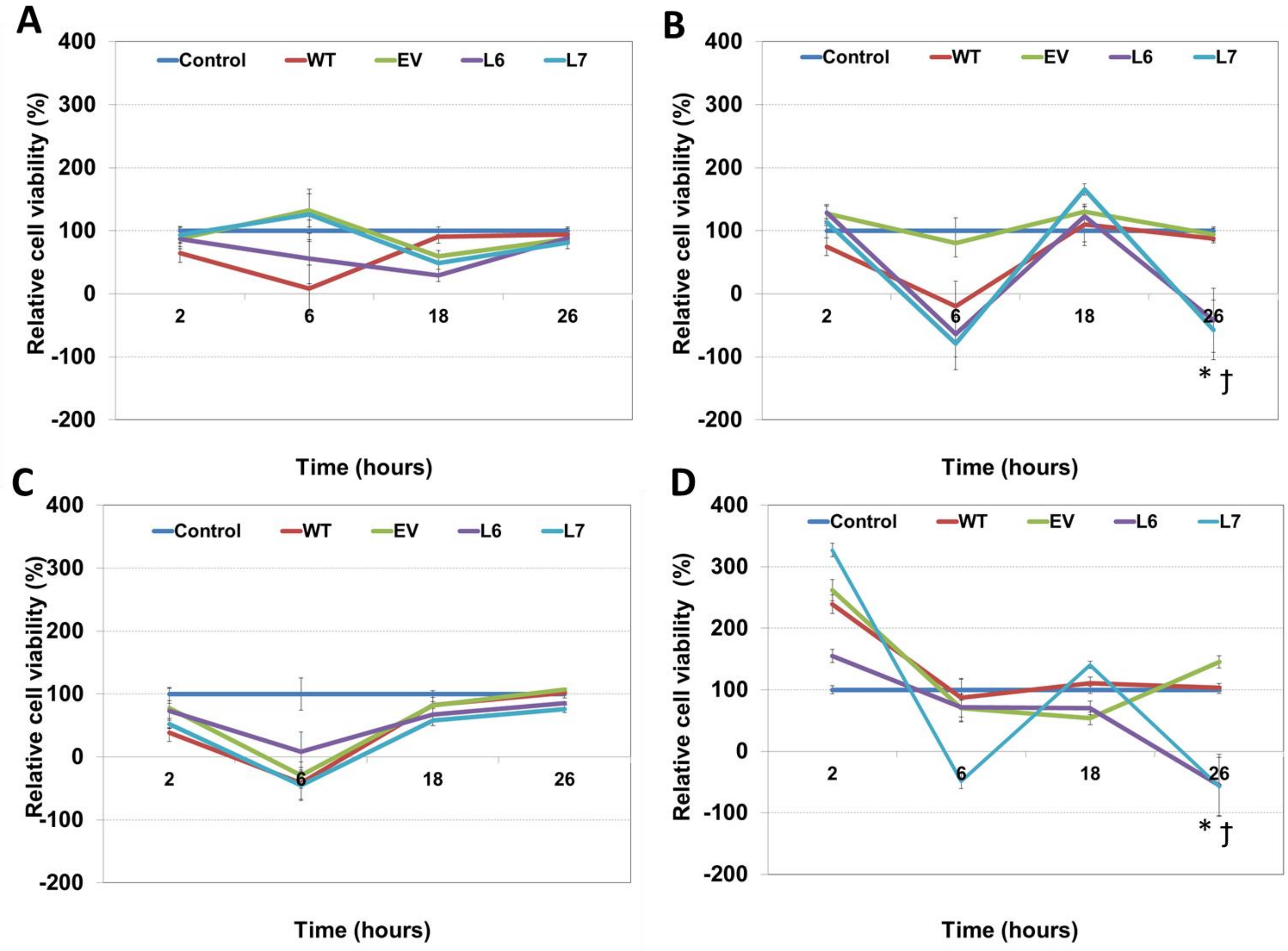

S1 Fig. MTT assay confirmation. The MCF-7 cells were incubated with different concentration (A: (0.1 µg/ml), B: (1 µg/ml), C: (2 µg/ml) and D: (3 µg/ml)) of the total metabolite extract of the control (WT, EV) and transgenic (L6, L7) tomato fruits. Samples were incubated for 2, 6, 18 and 26 hours of incubation after which were treated with MTT reagent. The absorbance was measured at 570 nm using a spectrophotometer. The cell viability was compared with non-treated cells. *, $p<0.05$ of L6 compared to control, ǂ, $p<0.05$ L7 compared to control.
