# Supplementary material for: Polyphenolic extract of InsP 5-ptase expressing tomato plants reduce the proliferation of MCF-7 breast cancer cells
Source: PLoS One. 2017 Apr 27;12(4):e0175778. doi: 10.1371/journal.pone.0175778 (PMC5407797; doi:10.1371/journal.pone.0175778)
Supplement: S2 Fig — qRT-PCR was performed for cyclin G1 and EGF-LIKE domain as representative of the genes with changes in expression level as indicated by microarray data. The RNA samples were isolated and the cDNA was synthesized from the control cells and those treated with an extract of wild-type or transgenic tomato fruits (lines 6 and 7). The results of the qRT-PCR for both genes confirm the up-regulation of the genes with changes similar to the microarray results. Methodological details: The transcript abundance was validated by using quantitative real-time PCR (qPCR) for two genes: Cyclin G1 and EGF-Like-Domain. The cDNA was synthesized by using SuperScript III First-Strand Synthesis System Kit (Invitrogen Inc.) and using Oligo(dT) primers. The synthesized cDNA was used as a template in the PCR reaction and gene-specific primers were used to amplify selected genes. The amplification of Cyclin G1 was carried by using a forward primer (5’- AGCTGAATGCCCTGTTGGAA -3’) and a reverse primer (5’- TGCAGTCATTCTGAGGCCAT -3’). The amplification of EGF-Like-Domain was carried by using a forward primer (5’- ATGAGACCGTCCTGGAGATGG -3’) and a reverse primer (5’- GCAGACCAGCCTCAGCAGAA -3’). The amplification of Actin was carried by using a forward primer (5’- TCGTCGCCCACATAGGAATC -3’) and a reverse primer (5’- TGCTCAGGGCTTCTTGTCCT -3’). The qRT-PCR analysis was carried using SYBR Green PCR master mix (Applied Biosystems, Inc.) using a C1000 Thermal Cycler equipped with a CFX96 Real-Time detection system (Bio-Rad, Hercules, CA, USA). The experiment was carried by using samples of three biological replicates and three technical replicates. Comparative count method was used to analyze and interpret the qPCR data. The extraction protocol was based on the method described by Ainsworth and Gillespie (2007) (1). The colorimetric assay works based on the transfer of electrons in alkaline medium from phenolic compounds to phosphomolybdic/ phosphotungstic acid complexes. This transfer is determined specifi [file pone.0175778.s002.pdf]

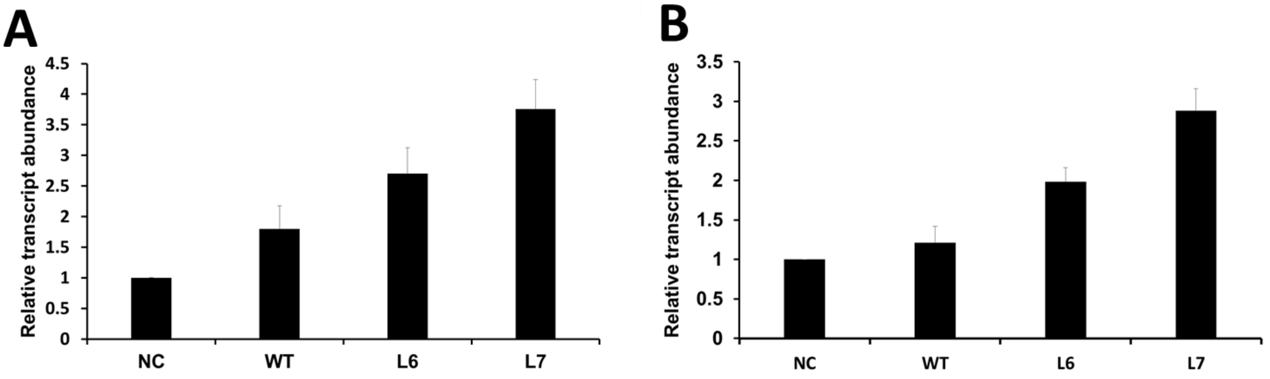


**S2 Fig**. **qRT-PCR confirmation of microarray data**. qRT-PCR was performed for cyclin G1 and EGF-LIKE domain as representative of the genes with changes in expression level as indicated by microarray data. The RNA samples were isolated and the cDNA was synthesized from the control cells and those treated with an extract of wild-type or transgenic tomato fruits (lines 6 and 7). The results of the qRT-PCR for both genes confirm the up-regulation of the genes with changes similar to the microarray results.

Methodological details:

The transcript abundance was validated by using quantitative real-time PCR (qPCR) for two genes: Cyclin G1 and EGF-Like-Domain. The cDNA was synthesized by using SuperScript III First-Strand Synthesis System Kit (Invitrogen Inc.) and using Oligo(dT) primers. The synthesized cDNA was used as a template in the PCR reaction and gene-specific primers were used to amplify selected genes. The amplification of Cyclin G1 was carried by using a forward primer (5'- AGCTGAATGCCCTGTTGGAA -3') and a reverse primer (5'-TGCAGTCATTCTGAGGCCAT -3'). The amplification of EGF-Like-Domain was carried by using a forward primer (5'- ATGAGACCGTCCTGGAGATGG -3') and a reverse primer (5'-GCAGACCAGCCTCAGCAGAA -3'). The amplification of Actin was carried by using a forward primer (5'- TCGTCGCCCACATAGGAATC -3') and a reverse primer (5'-TGCTCAGGGCTTCTTGTCCT -3'). The qRT-PCR analysis was carried using SYBR Green PCR master mix (Applied Biosystems, Inc.) using a C1000 Thermal Cycler equipped with a CFX96 Real-Time detection system (Bio-Rad, Hercules, CA, USA). The experiment was carried by using samples of three biological replicates and three technical replicates. Comparative count method was used to analyze and interpret the qPCR data. The extraction protocol was based on the method described by Ainsworth and Gillespie (2007) (1). The colorimetric assay works based on the transfer of electrons in alkaline medium from phenolic compounds to phosphomolybdic/ phosphotungstic acid complexes. This transfer is determined specifically at 765 nm by using spectrophotometry.
