# Supplementary material for: Polyphenolic extract of InsP 5-ptase expressing tomato plants reduce the proliferation of MCF-7 breast cancer cells
Source: PLoS One. 2017 Apr 27;12(4):e0175778. doi: 10.1371/journal.pone.0175778 (PMC5407797; doi:10.1371/journal.pone.0175778)
Supplement: S3 Fig — The major genes contributing to the highest impacted biological process was narrowed down using PANTHER classification software. (PDF) [file pone.0175778.s003.pdf]

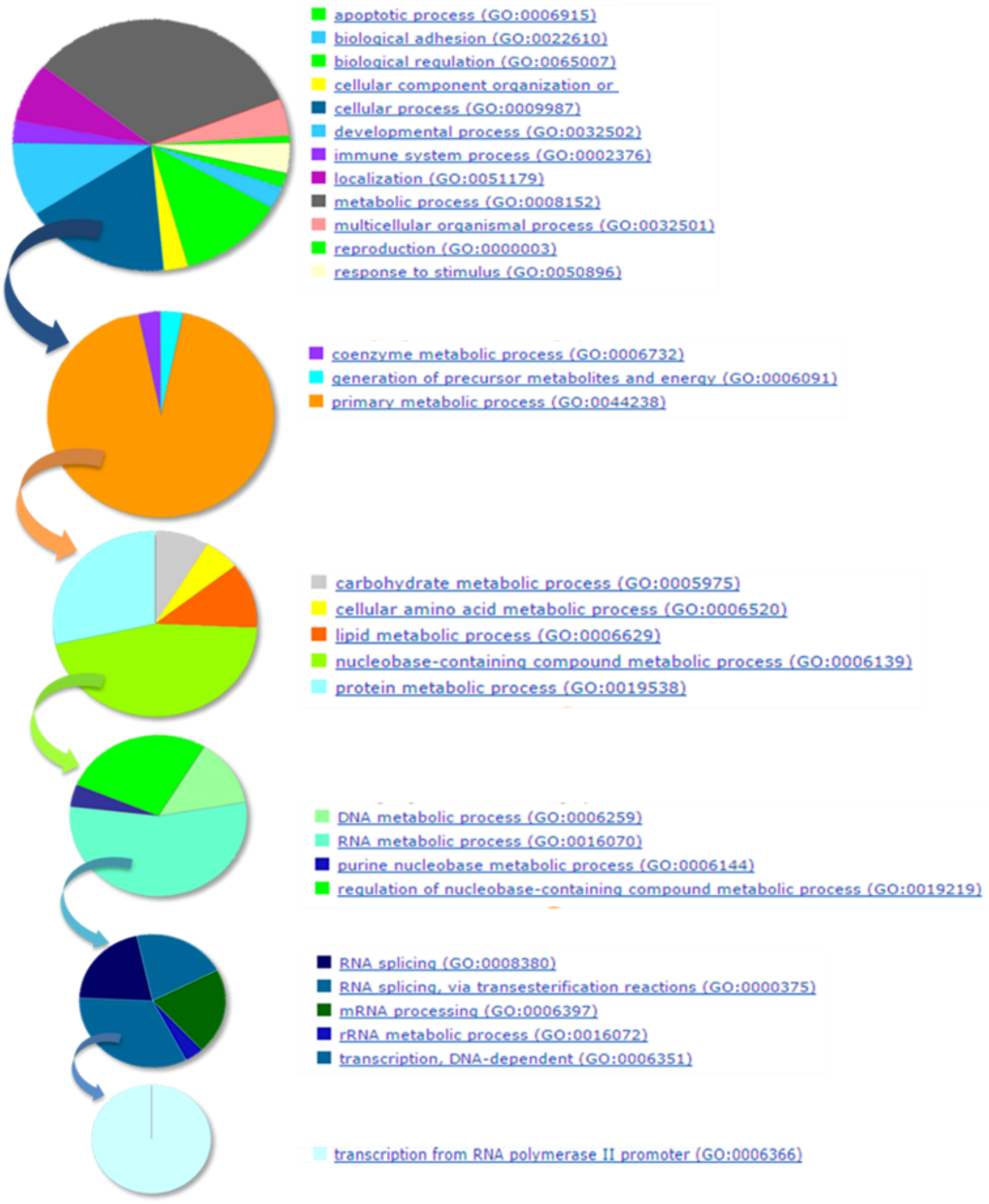

S3 Fig. Pie chart of main biological processes affected in MCF-7 cancer cell line by treatment with extract from InsP 5-ptase tomato fruits. The major genes contributing to the highest impacted biological process was narrowed down using PANTHER classification software
