# Supplementary material for: Polyphenolic extract of InsP 5-ptase expressing tomato plants reduce the proliferation of MCF-7 breast cancer cells
Source: PLoS One. 2017 Apr 27;12(4):e0175778. doi: 10.1371/journal.pone.0175778 (PMC5407797; doi:10.1371/journal.pone.0175778)
Supplement: S4 Fig — Raw data were converted to mzXML format by msConvert, were loaded into MZmine software for preprocessing. This software processes the raw data based on user-defined parameters specific to the machine used to collect data. A peak list for analysis was generated via mass detection, chromatogram building and deconvolution, normalization, alignment, and gap filling. Data were used to create the chromatogram and dendrogram seen in Fig 3A and 3B. Additionally, a list of over 7000 peaks that were suitable for comparison to the KEGG database was created for the initial identification of compounds. The KEGG database is one of the most comprehensive collections of metabolites to our knowledge. MZmine has an integrated search function that utilizes this database, which facilitates compound identification. Identification of metabolites is an ongoing challenge due to the lack of well-developed databases, especially for plants (45). Additionally, comparison to databases is generally unreliable due to the poor reproducibility of retention time for LC systems between laboratories. However, several successful attempts to identify the tomato metabolome by LC-MS have been reported, leading to the development of other databases, like MotoDB (http://www.ab.wur.nl/moto/) and KNApSAcK (http://kanaya.naist.jp/knapsack_jsp/top.html) (46,47). After initial compound identification, the generated peak list was uploaded to Metaboanalyst for statistical analysis. This software is hosted online and facilitates statistical analysis of data. It allows for normalization, sample group pooling, and data transformation andperforms a wide variety of statistical tests without requiring the user to learn any programming. Additionally, it allows for the development of visual interpretations of statistical analysis results, such as the one-way ANOVA in Fig 3C and the metabolite concentrations. The one-way ANOVA suggested over 2000 metabolites that were differently regulated between control and transgenic gr [file pone.0175778.s004.pdf]

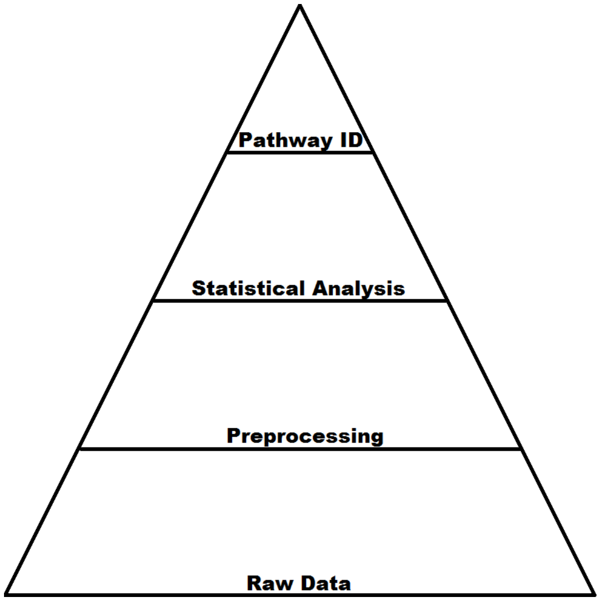

S4 Fig. **Overview of the metabolite analysis methodology.** Raw data were converted to mzXML format by msConvert, were loaded into MZmine software for preprocessing. This software processes the raw data based on user-defined parameters specific to the machine used to collect data. A peak list for analysis was generated via mass detection, chromatogram building and deconvolution, normalization, alignment, and gap filling. Data were used to create the chromatogram and dendrogram seen in Fig 3A and 3B. Additionally, a list of over 7000 peaks that were suitable for comparison to the KEGG database was created for the initial identification of compounds. The KEGG database is one of the most comprehensive collections of metabolites to our knowledge. MZmine has an integrated search function that utilizes this database, which facilitates compound identification. Identification of metabolites is an ongoing challenge due to the lack of well-developed databases, especially for plants (45). Additionally, comparison to databases is generally unreliable due to the poor reproducibility of retention time for LC systems between laboratories. However, several successful attempts to identify the tomato metabolome by LC-MS have been reported, leading to the development of other databases, like MotoDB (http://www.ab.wur.nl/moto/) and KNApSAcK (http://kanaya.naist.jp/knapsack_jsp/top.html) (46,47). After initial compound identification, the generated peak list was uploaded to Metaboanalyst for statistical analysis. This software is hosted online and facilitates statistical analysis of data. It allows for normalization, sample group pooling, and data transformation and performs a wide variety of statistical tests without requiring the user to learn any programming. Additionally, it allows for the development of visual interpretations of statistical analysis results, such as the one-way ANOVA in Figure 3C and the metabolite concentrations in Figure 4. The one-way ANOVA suggested over 2000 metabolites that were differently regulated between control and transgenic groups. Of these, Fisher's test identified 117 metabolites that were up-regulated in the transgenic lines. The up-regulated metabolites became the focus for a more in-depth, manual identification search using the KEGG database, KomicMarket database,

KNApSAck database, and publications (47,53,54). This search yielded 35 metabolites that could be putatively identified and were up-regulated, 11 of which had a flavonoid structure. Finally, the KEGG database was reference for pathway analysis using the cursorily identified up-regulated compounds seen in S2 Table
