# Supplementary material for: Polyphenolic extract of InsP 5-ptase expressing tomato plants reduce the proliferation of MCF-7 breast cancer cells
Source: PLoS One. 2017 Apr 27;12(4):e0175778. doi: 10.1371/journal.pone.0175778 (PMC5407797; doi:10.1371/journal.pone.0175778)
Supplement: S1 Table — 35 compounds have been tentatively identified by comparison to either a database or publication, 11 of which are flavonoids. Where the table lists KEGG as a source, MZmine software was used to compare the peak list to the database for identification purposes before the peak list was uploaded to Metaboanalyst, which was used for statistical analysis after MZmine completed processing the raw data. Not all compounds are in the KEGG database, but KEGG IDs are listed for those that were found. (PDF) [file pone.0175778.s005.pdf]

S1 Table. List of the compounds identified by Metaboanalyst software and were found significantly up-regulated in transgenic fruits of lines 6 and 7.

35 compounds have been tentatively identified by comparison to either a database or publication, 11 of which are flavonoids. Where the table lists KEGG as a source, MZmine software was used to compare the peak list to the database for identification purposes before the peak list was uploaded to Metaboanalyst, which was used for statistical analysis after MZmine completed processing the raw data. Not all compounds are in the KEGG database, but KEGG IDs are listed for those that were found.

m/z	RT (min)	KEGG ID	Tentative Identification	Source	Group
119.1066	28.6	C00601	Phenylacetylaldehyde	KEGG	aldehydes
128.1572	9.6	C01879	Pyroglutamic Acid	Gómez-Romero, Segura-Carretero, & Fernández-Gutiérrez, 2010	amino acids and derivatives
130.3659	10.7	C00123	Fructosyl leucine (-hexose)	Gómez-Romero, Segura-Carretero, & Fernández-Gutiérrez, 2010	amino acids and derivatives
151.1071	28.6	C07271	Limonene Oxide	Gómez-Romero, Segura-Carretero, & Fernández-Gutiérrez, 2010	terpenoids
170.8274	28.5				
267.2984	10.4	C02572	Feruloylquinic Acid	Gómez-Romero, Segura-Carretero, & Fernández-Gutiérrez, 2010	hydroxycinnamic acids
271.5263	28.6	C00509	Naringenin	Gómez-Romero, Segura-Carretero, & Fernández-Gutiérrez, 2010	flavonoids
278.8388	28.6	C01964	Tos-Ph-CH2Cl	KEGG	sulfonic acid derivative
278.8401	28.5				
278.8969	28.6				
290.5020	6.8		Fructosyl Glutamate (-H_2O)	Gómez-Romero, Segura-Carretero, & Fernández-Gutiérrez, 2010	amino acids and derivatives
292.7480	28.6		Fructosyl leucine	Gómez-Romero, Segura-Carretero, & Fernández-Gutiérrez, 2010	amino acids and derivatives
310.8639	28.6				

310.880	328.6				
311.005	128.6		Hydroperoxy-octadecanedioic acid	Gómez-Romero, Segura-Carretero, & Fernández-Gutiérrez, 2010	fatty acids
320.853	928.5				
320.897	628.7				
320.980	628.8	C00364	Thymidine 5'-phosphate	KEGG	nucleotides
324.855	928.6	C14967	1-[2-Bromo-1-(4-chlorophenyl)ethenyl]-2-chlorobenzene	KEGG	diarylmethane
324.914	028.6				
324.941	728.6				
365.547	516.8				
365.687	116.9				
367.256	510.5	C03309	Strictosidine aglycone	KEGG	alkaloids
368.679	828.5				
435.041	025.4	C01604	Phlorizin	KEGG	flavonoids
440.150	425.3	C00504	Folate	KEGG	vitamins
451.173	922.3	C07349	3'-Demethylstaurosporine	KEGG	alkaloids
452.758	74.3		Torvanol A	KNApSAck	hydrogen sulfate salt
455.021	522.3	C00061	Riboflavin-5-phosphate	KEGG	coenzymes
456.199	522.3	C00143	5,10-Methylenetetrahydrofolate	KEGG	coenzymes
456.394	522.2				
456.437	85.3				
456.778	122.3				
457.091	75.3	C01847	Reduced Flavin Mononucleotide	KEGG	coenzymes

464.8931	4.2				
464.9268	4.2				
499.4829	23.3				
499.8035	22.7				
499.9187	25.4				
500.0861	25.4				
500.1994	23.0				
500.1996	5.2				
500.2222	23.5				
500.3259	23.1				
500.4257	25.4				
500.6684	25.4				
500.7832	25.4				
501.7131	3.6				
514.6147	3.8				
554.8369	28.6				
554.8484	28.5				
558.6173	20.1				
564.7713	28.6				
565.7777	28.6				
566.5126	23.7				
566.7286	23.6				
567.1107	23.7				

593.1990	26.3	C03870	Isoorientin 2"-O-rhamnoside	KEGG	flavonoids
594.8683	28.5	C15641	XCT790	KEGG	cinnamamides
594.8764	19.6				
594.8854	19.7				
594.9083	19.7				
594.9254	28.4		Kaempferol 3-O-Rutinoside	KomicMarket	flavonoids
595.8089	21.3	C00509	Naringenin dihexose	Gómez-Romero, Segura-Carretero, & Fernández-Gutiérrez, 2010	flavonoids
597.0088	22.8				
597.2045	23.4	C00774	Phloretin Dihexoside	Gómez-Romero, Segura-Carretero, & Fernández-Gutiérrez, 2010	flavonoids
597.7015	22.5				
605.9548	5.0	C00043	UDP-N-acetyl-alpha-D-glucosamine	KEGG	nucleotides
606.9839	11.6				
609.6419	21.2		Rutin hexoside (-hexose)	Gómez-Romero, Segura-Carretero, & Fernández-Gutiérrez, 2010	flavonoids
609.7172	21.3		Kaempferol 3,7-dihexoside	Gómez-Romero, Segura-Carretero, & Fernández-Gutiérrez, 2010	flavonoids
610.5469	21.2				
610.9077	26.1				
611.4571	21.1				
611.4803	21.1				
611.9915	26.1				
612.3288	21.1				

614.3140	22.0				
614.3481	22.1				
614.8316	4.8				
622.8930	28.5				
622.9337	28.6				
622.9401	28.6				
624.8898	20.2				
624.9094	20.2				
626.9210	23.3				
626.9905	23.4				
630.8975	19.5				
630.9204	19.5				
630.9501	19.4				
630.9710	20.5	C19971	UDP-2,4-bis(acetamido)-2,4,6-trideoxy-beta-L-altropyranose	KEGG	nucleotides
635.1261	28.3				
635.1592	28.3	C04360	1-O,2-O,6-O-Trigalloyl-beta-D-glucose	KEGG	tannins
635.2334	28.2				
637.7765	24.6				
637.9276	24.8	C12632	Luteolin 7-O-beta-D-diglucuronide	KEGG	flavonoids
638.5831	24.9				
638.6937	24.8				
639.1892	23.5	C10084	Isorhamnetin 3-sophoroside	Gómez-Romero, Segura-Carretero, & Fernández-Gutiérrez, 2010	flavonoids

646.9345	21.1				
647.0235	21.1				
652.8937	15.0				
652.8970	15.1				
653.0002	22.1		Cilistol p	KNApSAck	steroid glycoside
653.0365	22.1				
653.5294	27.5	C05811	3-Octoprenylcatechol	KEGG	flavonoids
655.1387	25.4				
655.1842	25.3	C10466	Hellicoside	KEGG	hydroxycinnamic acids
660.8475	20.6				
680.8227	24.7				
680.8809	24.9				
682.9061	24.5				
754.8743	21.4				
755.8232	21.4				
758.8626	21.0				
758.8742	21.0				
