# Supplementary material for: Polyphenolic extract of InsP 5-ptase expressing tomato plants reduce the proliferation of MCF-7 breast cancer cells
Source: PLoS One. 2017 Apr 27;12(4):e0175778. doi: 10.1371/journal.pone.0175778 (PMC5407797; doi:10.1371/journal.pone.0175778)
Supplement: S2 Table — The list of KEGG IDs from S1 Table was entered into the KEGG Pathway search that resulted in the 35 tentatively identified compounds that were up-regulated in both transgenic lines (6, 7) and their associated pathways, if available. The most common pathways were metabolic and biosynthesis of secondary metabolites. The most common type of identified compound was in the flavonoid category. (PDF) [file pone.0175778.s006.pdf]

S2 Table. Identified up-regulated compounds in transgenic tomato fruits. The list of KEGG IDs from S1 Table was entered into the KEGG Pathway search that resulted in the 35 tentatively identified compounds that were up-regulated in both transgenic lines (6, 7) and their associated pathways, if available. The most common pathways were metabolic and biosynthesis of secondary metabolites. The most common type of identified compound was in the flavonoid category.

m/z	RT (min)	KEGG ID	Tentative Identification	Source	Group	KEGG Pathways
119.1066	28.6	C00601	Phenylacetylaldehyde	KEGG	aldehydes	Metabolic pathways, Phenylalanine metabolism
128.1572	9.6	C01879	Pyroglutamic Acid	Gómez-Romero, Segura-Carretero, & Fernández-Gutiérrez, 2010	amino acids and derivatives	Glutathione metabolism
130.3659	10.7	C00123	Fructosyl leucine (-hexose)	Gómez-Romero, Segura-Carretero, & Fernández-Gutiérrez, 2010	amino acids and derivatives	Metabolic pathways, Biosynthesis of secondary metabolites, ABC transporters, Valine, leucine and isoleucine degradation, Aminoacyl-tRNA biosynthesis, Valine, leucine and isoleucine biosynthesis, Glucosinolate biosynthesis, Biosynthesis of amino acids, 2-Oxocarboxylic acid metabolism
151.1071	28.6	C07271	Limonene Oxide	Gómez-Romero, Segura-Carretero, & Fernández-Gutiérrez, 2010	terpenoids	Limonene and pinene degradation
267.2984	10.4	C02572	Feruloylquinic Acid	Gómez-Romero, Segura-	hydroxycinnamic acids	

				Carretero, & Fernández-Gutiérrez, 2010		
271.5263	28.6	C00509	Naringenin	Gómez-Romero, Segura-Carretero, & Fernández-Gutiérrez, 2010	flavonoids	Metabolic pathways, Biosynthesis of secondary metabolites, Flavonoid biosynthesis
278.8388	28.6	C01964	Tos-Ph-CH2Cl	KEGG	sulfonic acid derivative	
290.5020	6.8		Fructosyl Glutamate (-H_2O)	Gómez-Romero, Segura-Carretero, & Fernández-Gutiérrez, 2010	amino acids and derivatives	
292.7480	28.6		Fructosyl leucine	Gómez-Romero, Segura-Carretero, & Fernández-Gutiérrez, 2010	amino acids and derivatives	
311.0051	28.6		Hydroperoxy-octadecanedioic acid	Gómez-Romero, Segura-Carretero, & Fernández-Gutiérrez, 2010	fatty acids	

320.9806	28.8	C00364	Thymidine 5'-phosphate	KEGG	nucleotides	Metabolic pathways, Pyrimidine metabolism
324.8559	28.6	C14967	1-[2-Bromo-1-(4-chlorophenyl)ethenyl]-2-chlorobenzene	KEGG	diarylmethane	
367.2565	10.5	C03309	Strictosidine aglycone	KEGG	alkaloids	Metabolic pathways, Biosynthesis of secondary metabolites
435.0410	25.4	C01604	Phlorizin	KEGG	flavonoids	
440.1504	25.3	C00504	Folate	KEGG	vitamins	Metabolic pathways, One carbon pool by folate, Folate biosynthesis
451.1739	22.3	C07349	3'-Demethylstaurosporine	KEGG	alkaloids	
452.7587	4.3		Torvanol A	KNApSAck	hydrogen sulfate salt	
455.0215	22.3	C00061	Riboflavin-5-phosphate	KEGG	coenzymes	Metabolic pathways, Biosynthesis of secondary metabolites, Riboflavin metabolism, Oxidative phosphorylation
456.1995	22.3	C00143	5,10-Methylenetetrahydrofolate	KEGG	coenzymes	Metabolic pathways, One carbon pool by folate, Carbon metabolism, Glycine, serine and threonine metabolism
457.0917	5.3	C01847	Reduced Flavin Mononucleotide	KEGG	coenzymes	
593.1990	26.3	C03870	Isoorientin 2"-O-rhamnoside	KEGG	flavonoids	Metabolic pathways, Riboflavin metabolism
594.8683	28.5	C15641	XCT790	KEGG	cinnamamides	
594.9254	28.4		Kaempferol 3-O-Rutinoside	KomicMarket	flavonoids	
595.8089	21.3	C00509	Naringenin dihexose	Gómez-Romero, Segura-Carretero, & Fernández-Gutiérrez,	flavonoids	

				2010		
597.2045	23.4	C00774	Phloretin Dihexoside	Gómez-Romero, Segura-Carretero, & Fernández-Gutiérrez, 2010	flavonoids	
605.9548	5.0	C00043	UDP-N-acetyl-alpha-D-glucosamine	KEGG	nucleotides	Amino sugar and nucleotide sugar metabolism, Vancomycin resistance, Insulin resistance, Metabolic pathways
609.6419	21.2		Rutin hexoside (-hexose)	Gómez-Romero, Segura-Carretero, & Fernández-Gutiérrez, 2010	flavonoids	
609.7172	21.3		Kaempferol 3,7-dihexoside	Gómez-Romero, Segura-Carretero, & Fernández-Gutiérrez, 2010	flavonoids	
630.9710	20.5	C19971	UDP-2,4-bis(acetamido)-2,4,6-trideoxy-beta-L-altropyranose	KEGG	nucleotides	Amino sugar and nucleotide sugar metabolism
635.1592	28.3	C04360	1-O,2-O,6-O-Trigalloyl-beta-D-glucose	KEGG	tannins	
637.9276	24.8	C12632	Luteolin 7-O-beta-D-diglucuronide	KEGG	flavonoids	Flavone and flavonol biosynthesis
639.1892	23.5	C10084	Isorhamnetin 3-sophoroside	Gómez-Romero,	flavonoids	

				Segura-Carretero, & Fernández-Gutiérrez, 2010		
653.0002	22.1		Cilistol p	KNApSAck	steroid glycoside	
653.5294	27.5	C05811	3-Octoprenylcatechol	KEGG	flavonoids	Biosynthesis of secondary metabolites, Ubiquinone and other terpenoid-quinone biosythesis
655.1842	25.3	C10466	Hellicoside	KEGG	hydroxycinnamic acids	
