# Supplementary material for: Polyphenolic extract of InsP 5-ptase expressing tomato plants reduce the proliferation of MCF-7 breast cancer cells
Source: PLoS One. 2017 Apr 27;12(4):e0175778. doi: 10.1371/journal.pone.0175778 (PMC5407797; doi:10.1371/journal.pone.0175778)
Supplement: S3 Table — The genes labeled in red are genes that were up-regulated in the cells after treatment with total metabolite extract of transgenic fruits (L6 and L7) and genes labeled green are the genes that were down-regulated after the same treatment. (PDF) [file pone.0175778.s007.pdf]

S3 Table. List of genes involved in transcription of RNA polymerase II promoter found by using PANTHER classification software analysis. The genes labeled in red are genes that were up-regulated in the cells after treatment with total metabolite extract of transgenic fruits (L6 and L7) and genes labeled green are the genes that were down-regulated after the same treatment.

	Gene ID	Gene Name	Gene Symbol	ortholog	PANTHER Protein Class	PANTHER Family/Subfamily	
1	10322	SET and MYND domain-containing protein 5	SMYD5	ortholog	SET AND MYND DOMAIN-CONTAINING PROTEIN 5 (PTHR12197:SF153)	transcription cofactor	Homo sapiens
2	23613	Protein kinase C-binding protein 1	ZMYND8	ortholog	PROTEIN KINASE C-BINDING PROTEIN 1 (PTHR24102:SF13)	transcription cofactor ubiquitin-protein ligase	Homo sapiens
3	4089	Mothers against decapentaplegic homolog 4	SMAD4	ortholog	MOTHERS AGAINST DECAPENTAPLEGIC HOMOLOG 4 (PTHR13703:SF19)	transcription factor	Homo sapiens
4	155054	Zinc finger protein 425	ZNF425	ortholog	ZINC FINGER PROTEIN 425 (PTHR24381:SF120)	KRAB box transcription factor	Homo sapiens
5	4212	Homeobox protein Meis2	MEIS2	ortholog	HOMEOBOX PROTEIN MEIS2 (PTHR11850:SF47)	DNA-directed RNA polymerase	Homo sapiens
6	26986	Polyadenylate-binding protein 1	PABPC1	ortholog	POLYADENYLATE-BINDING PROTEIN 1 (PTHR24011:SF233)	transcription factor<br>DNA binding protein<br>mRNA polyadenylation factor<br>mRNA splicing factor<br>ribonucleoprotein	Homo sapiens
7	3670	Insulin gene enhancer protein ISL-1	ISL1	ortholog	INSULIN GENE ENHANCER PROTEIN ISL-1 (PTHR24204:SF3)	homeobox transcription factor<br>zinc finger transcription factor	Homo sapiens
8	219749	Zinc finger protein 25	ZNF25	ortholog	ZINC FINGER PROTEIN 25 (PTHR24381:SF62)	KRAB box transcription factor	Homo sapiens
